# Modeling the role of fertilizers and improved seeds on rice productivity in Tanzania using a stochastic simulation approach

**DOI:** 10.1371/journal.pone.0353377

**Published:** 2026-08-26

**Authors:** Ibrahim L. Kadigi, Lazaro Kagata, Stefan Sieber

**Affiliations:** 1 Department of Business Management, College of Humanities and Business Studies, Mbeya University of Science and Technology (MUST), Mbeya, Tanzania; 2 Leibniz-Zentrum fur Agrarlandschaftsforschung (ZALF e. V.), Müncheberg, Germany; 3 Department of Agricultural Economics, Faculty of Life Sciences Thaer-Institute, Humboldt-Universität zu Berlin, Berlin, Germany; National Museums of Kenya, KENYA

## Abstract

Climate change poses a significant threat to food production in Tanzania, particularly for smallholder farmers who rely on rain-fed agriculture. This study examines the role of improved seeds and fertilizers as climate adaptation strategies to boost rice productivity and food security. Using data from the 2019/20 National Sample Census of Agriculture (NSCA), the study analyzed 6,025 rice farms from Mainland Tanzania and Zanzibar. A Stochastic Simulation model was used to account for yield variability and uncertainty. Farms were classified by seed type (local or improved) and fertilizer use (organic, inorganic, or none). Findings show that using improved seeds with inorganic fertilizers significantly enhances productivity, with a 16% chance of achieving yields above 4.5 t/ha. This combination also reduced the risk of falling below food security thresholds. In contrast, farms using local seeds, even with fertilizers, had only a 10% chance of reaching that yield level. The impact was more notable in Mainland Tanzania (25%) than in Zanzibar (6%). While organic fertilizers improve soil health, they offer limited short-term yield gains. The study concludes that promoting access to improved seeds and inorganic fertilizers is crucial for enhancing rice productivity and achieving the SDG 2 goal of Zero Hunger in Tanzania.

## Introduction

Climate change poses a significant and growing threat to global food security, with particularly severe consequences for smallholder farming systems in sub-Saharan Africa that depend largely on rain-fed agriculture. Increasing temperature extremes, erratic rainfall patterns, and the rising frequency of droughts and floods have intensified yield variability and production risk, undermining the stability of food systems and rural livelihoods [1–7]. In Tanzania, these climatic pressures are compounded by limited access to modern agricultural inputs, low levels of mechanization, and persistent yield gaps, particularly among smallholder rice farmers [8–12].

Rice is a strategic staple crop in Tanzania, contributing significantly to national food security, rural employment, and household income. Demand for rice has grown rapidly due to population growth, urbanization, and dietary transitions, yet average yields remain substantially below attainable levels compared with other major rice-producing regions [13–16]. National policy frameworks and development strategies, including Tanzania’s agricultural transformation agenda and the Tanzania Seed Sector Development Strategy [17], consistently emphasize the promotion of improved seed varieties and fertilizer use as key pathways for closing yield gaps, stabilizing production, and achieving Sustainable Development Goal 2 (Zero Hunger) [9,10,18–22].

A substantial body of empirical literature documents that improved seed varieties enhance yield potential, stress tolerance, and responsiveness to nutrients, while fertilizer use supplies essential macro- and micronutrients required to realize this potential [15,23–25]. Studies further distinguish between the short-term productivity gains associated with inorganic fertilizers and the longer-term soil health and sustainability benefits of organic fertilizers and integrated nutrient management approaches [26–29]. Recent evidence from sub-Saharan Africa suggests that combining improved seeds with appropriate fertilizer regimes can substantially reduce yield gaps, although outcomes vary across agroecological zones and management contexts [15,18,25,30].

Despite this extensive literature, most existing studies evaluate the impacts of seed and fertilizer use based on mean-based indicators of productivity or focus on the socioeconomic determinants of adoption (e.g., access to credit, extension services, and markets). While such analyses are valuable, they provide limited insight into production risk, yield uncertainty, and food security outcomes, which are central concerns for smallholder households operating in highly variable, climate-sensitive environments [13,31,32]. In practice, farmers and policymakers are often less concerned with average yields than with the likelihood of yield gaps or harvest shortfalls that can potentially jeopardize food security or depress projected profit margins.

From a methodological standpoint, traditional econometric approaches such as ordinary least squares or generalized linear models are well-suited for estimating conditional mean effects. Still, they are less informative for evaluating downside risk and the probability of extreme outcomes. Smallholder rice yields in Tanzania are highly heterogeneous, often non-normally distributed, and strongly influenced by agroecological variability and management differences [2,11]. Stochastic and probabilistic simulation methods, particularly Monte Carlo Simulation (MCS), are increasingly used in agricultural economics and risk analysis to address these challenges by modeling full outcome distributions rather than single-point estimates [33–35]. Such approaches allow explicit estimation of the probability that yields fall below critical food-security thresholds or exceed aspirational productivity targets, making them especially relevant for policy analysis under uncertainty [1,4].

However, nationally representative applications of stochastic simulation to evaluate alternative seed–fertilizer pathways in Tanzania’s rice sector remain limited. Existing studies often rely on experimental plots, localized surveys, or adoption models without linking observed management practices to risk-adjusted food-security outcomes across regions and agroecological zones [12,16]. Consequently, there is insufficient evidence on how different combinations of local versus improved seeds and organic versus inorganic fertilizers translate into the probability of achieving food-security-relevant yield targets at scale.

This study addresses this gap by applying a stochastic simulation framework to nationally representative data from the 2019/20 National Sample Census of Agriculture (NSCA). By modeling yield distributions for alternative seed–fertilizer combinations and evaluating their probabilities relative to minimum and maximum food security thresholds, the study advances knowledge beyond mean-based analyses. Specifically, the central research question guiding this analysis is: *What is the likelihood that a given seed–fertilizer practice enables rice-farming households to exceed an upper yield target while avoiding yields below a minimum food-security threshold under conditions of production uncertainty?*

By answering this question for Mainland Tanzania, Zanzibar, and major rice-producing agroecological zones, the study provides policy-relevant evidence on the relative merits of alternative input strategies. By answering this question and filling the knowledge gap, this study pursues three objectives: (1) to estimate and compare stochastic yield distributions for rice farming practices defined by seed type and fertilizer use; (2) to quantify and rank the probabilities that each practice exceeds an upper yield target (4.5 t/ha) and avoids falling below a minimum food-security threshold (2.0 t/ha) across Mainland Tanzania, Zanzibar, and major rice-producing agroecological zones; and (3) to statistically assess whether yield distributions under improved-input practices differ significantly from the baseline practice (local seeds without fertilizer). In doing so, it contributes to the design of targeted, risk-informed interventions to enhance rice productivity, stabilize food supplies, and support Tanzania’s progress toward SDG 2 (Zero Hunger).

## Materials and methods

### Study area

This study focuses on rice farms from both mainland Tanzania and Zanzibar. The study also delves deeper into the two major rice agroecological zones: the eastern zone (mainly the Morogoro region) and the southern highlands zone (mainly the Mbeya region). These zones are characterized by distinct climatic patterns, soil types, and farming systems, significantly shaping rice productivity in Tanzania. By grouping rice farms into specific agroecological zones, this research enables a detailed examination of how farming practices, particularly fertilizer and seed use, perform under varied ecological conditions. This approach ensures that the study’s findings are representative and practical for implementation across the country and within the major rice-producing regions, benefiting farmers, agricultural policymakers, and development agencies.

The division into agroecological zones facilitates a comparative analysis of farming practices, highlighting their effectiveness in different environmental contexts. Such comparisons are crucial for developing region-specific strategies that optimize the use of agricultural inputs and enhance crop yields. Moreover, analyzing how each zone responds to climatic and environmental challenges allows for better planning and the development of climate-resilient farming systems. This level of detail supports the creation of localized policies that address region-specific constraints, ultimately contributing to sustainable and adaptive agricultural growth in Tanzania.

The six agroecological zones selected for this study include:

i Rice farms in Mainland Tanzania, including all regions: Kagera, Mara, Mwanza, Simiyu, Geita, Shinyanga, Kigoma, Tabora, Arusha, Manyara, Kilimanjaro, Tanga, Dodoma, Singida, Katavi, Kigoma, Rukwa, Mbeya, Songwe, Njombe, Iringa, Morogoro, Lindi, Pwani, Dar-es-salaam, Ruvuma, Mtwara.
ii Rice farms in Zanzibar (Mjini Magharibi, Kusini Unguja, Kaskazini Unguja).
iii Rice farms in the Eastern Zone (EZ), mainly in the Morogoro region.
iv Rice farms in the Southern Highlands Zone (SHZ), mainly in the Mbeya region.

This classification captures Tanzania’s agricultural heterogeneity, enabling a robust analysis of productivity trends across ecological zones. The study identifies the most effective interventions to improve rice yields by considering these variations. It supports evidence-based policy decisions to close the productivity gap in Tanzania’s rice production.

### Data sources

Tanzania has conducted five National Sample Censuses of Agriculture (NSCA) as of the 2019/2020 census, marking the most recent; the first census took place in 1971/1972, followed by the second in 1994/1995, the third in 2002/2003, and the fourth in 2007/2008. This study primarily uses data from the 2019/20 National Sample Census of Agriculture (NSCA), which is the most recent nationally representative agricultural census conducted in Tanzania. The census was implemented by the Tanzania National Bureau of Statistics (NBS) in collaboration with the World Bank, under the authority of the United Republic of Tanzania, and provides comprehensive, up-to-date information on crop production, input use, and farm characteristics [36]. The World Bank also plays a significant role in the NSCA data collection process, primarily by providing technical assistance, funding, and logistical support rather than serving as the direct field collector. The immediately preceding 2007/08 NSCA was used only as a historical reference to normalize and adjust the 2019/20 yield distributions within the stochastic simulation framework, thereby improving temporal comparability without substituting current production conditions. The 2019/20 NSCA employed a two-stage sampling design covering 2,820 Primary Sampling Units (PSUs), of which 2,670 were located in Mainland Tanzania and 150 in Zanzibar. From these PSUs, a total of 6,145 rice-producing households were identified and included in this analysis, comprising 5,536 households in Mainland Tanzania and 609 in Zanzibar. The NSCA serves as a comprehensive repository of information, capturing key aspects of agricultural households, including farm size, crop production, livestock holdings, and the use of agricultural inputs such as fertilizers and seeds. Additionally, the census examines rural infrastructure improvements and the living standards of farming households, offering valuable benchmarks for evaluating agricultural productivity and the impact of agricultural interventions implemented by various stakeholders, including Agricultural Sector Lead Ministries (ASLMs).

To ensure nationwide representativeness, the NSCA employed a two-stage sampling methodology. In the first stage, Census Enumeration Areas (CEAs) from the 2012 Population and Housing Census were systematically selected as Primary Sampling Units (PSUs). In the NSCA sampling design, PSUs were allocated proportionally across regions and districts based on the distribution of agricultural households in the national sampling frame. This proportional allocation ensured that areas with higher agricultural activity contributed a larger share of the sample. PSUs and households within PSUs were then systematically selected using an interval-based procedure with a random start, ensuring spatially even coverage and reducing selection bias. Together, these procedures underpin the national and regional representativeness of the data used in this study. In the second stage, agricultural households within the selected PSUs were randomly sampled, with a focus on households actively engaged in crop cultivation and livestock production.

The sampling process covered a total of 2,820 PSUs, comprising 2,670 PSUs from Mainland Tanzania and 150 PSUs from Tanzania Zanzibar. The sampling strategy employed a Probability Proportional to Size (PPS) technique, which prioritizes areas with higher agricultural activity, thereby enhancing the reliability and accuracy of the data. Details of the sampling procedure, including design and implementation, can be found in the official NSCA report [36]. The NSCA dataset used in this study focuses primarily on crop production and input use and does not collect detailed livestock production variables suitable for integrated crop–livestock system analysis. Consequently, all households identified as rice producers within the sampled Primary Sampling Units (PSUs) were included in the analysis, irrespective of livestock ownership, and no livestock-based sampling or filtering criteria were applied.

### Data processing and simulation

The first stage of the analysis involved organizing the NSCA production data by systematically classifying farms according to both farming practices and location: Mainland Tanzania, Zanzibar, and the two major rice-producing agroecological zones (AEZs): the Eastern Zone and the Southern Highlands Zone. Farms were categorized into six distinct farming practices based on seed type and fertilizer application. The baseline category, denoted as *F1.0*, comprises farms using local seeds without fertilizer. Alternative practices include *F1.1* and F1.2, representing farms using local seeds combined with organic and inorganic fertilizers, respectively. Similarly, *F2.0*, *F2.1*, and *F2.2* denote farms using improved seeds without fertilizer, with organic fertilizers, and with inorganic fertilizers, respectively. Table 1 presents the distribution of observations across these farming practices for both Mainland Tanzania and Zanzibar, as classified in the NSCA dataset. Further details on the simulation procedures applied to these categorized data are provided in subsequent sections.

**Table 1:** Number of observations for rice farms in mainland Tanzania and Zanzibar under different fertilizer and seed types farming practices.

Farming practices	Mainland Tanzania			Zanzibar	Total
	Farms in mainland TZ	Farms in Morogoro	Farms in Mbeya	Farms in Zanzibar	All TZ farms
Farming Practice 1 (*F1.0*)	4,073	768	369	179	4,252
Farming Practice 2 (*F1.1*)	646	100	204	132	778
Farming Practice 3 (*F1.2*)	581	95	187	123	704
Farming Practice 4 (*F2.0*)	129	28	22	58	187
Farming Practice 5 (*F2.1*)	6	–	–	–	6
Farming Practice 6 (*F2.2*)	101	15	59	117	218
**Total**	**5,536**	**1,006**	**841**	**609**	**6,145**

***Note:*** *FP1.0 = local seeds without fertilizers; F1.1 = local seeds with organic fertilizers; F1.2 = local seeds with inorganic fertilizers; F2.0 = improved seeds without fertilizer; F2.1 = improved seeds with organic fertilizers; F2.2 = improved seeds with inorganic fertilizers.*

### Deterministic yield computation

Before simulation, for each farming practice (defined in Table 1), all household-level rice production data were converted into yield per hectare (t/ha) to ensure unit consistency and comparability across farms. The analysis focuses exclusively on rice crop production; livestock variables were not included. Data were stratified by seed type, fertilizer application, and spatial unit, and observed yields were then transformed into fractional deviations from their group-specific means. These dimensionless deviations served as the empirical basis for a Probabilistic Simulation Approach (PSA) under Monte Carlo Simulation procedures and were rescaled to yield levels during model execution, ensuring consistency of scale across farming systems and agroecological zones [21,22].

Equation (1) is the deterministic yield, which was calculated as the ratio of total tonnage harvested to the area harvested (in hectares):


$$\begin{array}{c} {\;yield\;(\bar{y}}_{c,i})=\frac{Tones\;harvested\;(tones)}{Hectares\;harvested\;(a)} \end{array}$$ (1)

This step provided the base mean yield values per hectare for each farming practice at each farm location, laying the foundation for subsequent stochastic simulations.

### Stochastic yield simulation using the Monte Carlo simulation protocol

Since we have several independent random yields from both the Tanzania mainland, Zanzibar, and selected AEZs, with different numbers of observations, a Univariate Empirical (UVE) was first parameterized. The UVA was used because it enables probabilistic simulation, which is the study's principal research question. The UVE was simulated to develop an independent, uncorrelated sample size of 500 for each farming practice. The deterministic yield computed in Equation 1 was converted into stochastic form (Equation 2), where a random shock factor (ε) was introduced to capture unexplained yield variability. In the stochastic simulation model, household sample size is incorporated implicitly through the empirical yield distributions constructed for each farming practice and region. Specifically, the deterministic mean yield (${\bar{y}}_{i}$) per each farming system *i* and the empirical distribution of fractional deviations from the mean ($S_{y,i}$) are derived from observed household-level yields within each PSU and agroecological zone and form the basis for the Monte Carlo simulation.

Unlike approaches that primarily estimate mean yield differences under strong parametric assumptions, MCS reproduces the full yield distribution, allowing explicit characterization of downside risk and upside potential. This is particularly important for smallholder rice systems where yield outcomes are heterogeneous, often non-normal, and sensitive to agroecological conditions and management variability. Equation 3 expresses how the stochastic yields were simulated. The equation uses the Simetar function (***EMP()***) to simulate yields, resulting in a percentage deviation from the mean.


$$\begin{array}{c} {\tilde{y}}_{i}={\;\bar{y}}_{i}*\varepsilon \end{array}$$ (2)

Where the ε is a random shock factor expressed as $y_{i}-{\bar{y}}_{i}$


$$\begin{array}{c} {\tilde{y}}_{i}={\bar{y}}_{i}*\left(\text{1}+EMP\left(S_{y,i},P\left(S_{y,i}\right),ISND_{y,i}\right)\right)*\beta_{{\tilde{y}}_{\text{0}}} \end{array}$$ (3)

Where:

~ = A tilde represents a randomness (stochastic) variable.

*i* =Type of farming practices used (*F1.0, F1.1, F1.2, F2.0, F2.1* and *F2.2*).

*a_i_* = Hectares (ha) allocated for farming practice *i*

${\tilde{y}}_{i}$ = Stochastic mean yield per ha for farming practice *i*

${\overset{―}{y}}_{i}$ = Deterministic (mean) yield per ha for farming practice *i*

$\beta_{{\tilde{y}}_{\text{0}}}$ = The normalization factor, which is given by $\frac{{\tilde{y}}_{c,i}}{{\tilde{y}}_{h,i}}\;$and it is used to scale/adjust the 2019/20 NCSA mean yield.

${\tilde{y}}_{c}$ = Stochastic yield for the current survey (2019/20 NCSA)

${\tilde{y}}_{h}$ = Stochastic yield for the historical or previous survey (2007/08 NCSA)

*S*_*y*_ = Fraction deviations from the mean or sorted array of random yields for farming practice *i*

*P(S*_*y*_*) =* Cumulative probability function for the *S*_*y*_ values

*ISND*_*y*_ = Simetar function to simulate the independent standard normal deviates.

*EMP() =* Simetar function used to simulate a stochastic variable (yield)

The Independent Standard Normal Deviate (ISND) function was incorporated into the UVE model to introduce a source of stochastic variability into the simulation outputs. This function allows the simulated values to reflect the probabilistic uncertainty inherent in the empirical distribution of the variable being modeled. The ISND is typically denoted as ε ~ N(0, 1), which implies a standard normal distribution with a mean of zero (0) and a standard deviation of one (1). It operates independently of other random draws or variables in the simulation, ensuring uncorrelated randomness in each iteration. To generate a sufficiently large and representative sample for each farming practice, Equation (3) was simulated using the Latin Hypercube Sampling (LHS) technique within the Simetar software environment. The procedure followed the guidelines established by Richardson et al. [33] and extended by Rezende & Richardson [37] and Kadigi et al. [20,35]. The LHS method was used due to its efficiency in producing representative samples from complex probability distributions while requiring relatively few simulation runs. In this study, 500 iterations were conducted per farming system, a sample size considered adequate to capture the key statistical characteristics of the parent yield distributions.

A crucial component of the analytical process was model validation. Simulated yields generated from the UVE model were systematically compared to historical observations using statistical goodness-of-fit tests and probability density functions (PDFs). This comparison aimed to verify the consistency between the empirical and simulated yield distributions. The validation results confirmed that the stochastic model accurately replicated both the magnitude and variability of observed yields while preserving the underlying probabilistic structure introduced through Monte Carlo sampling. This alignment between simulated and actual data reinforces the model's reliability, confirming that the simulated outcomes are representative, unbiased, and robust enough to evaluate productivity across Tanzania’s diverse agroecological zones.

### Correlated uniform standard deviation (CUSD) of random yields

Following the validation of univariate simulations, a more robust multivariate model was developed using the Multivariate Empirical (MVE) distribution. This method used UVE output (simulated samples from 500 iterations per farming system) to estimate yield outcomes while accounting for interdependencies across farming practices per regional and AEZs. The process began with the calculation of residuals, representing percentage deviations of individual yield values from their respective means. Again, these deviations were derived using the Empirical function (EMP), which facilitated the parameterization of the MVE distribution. The residuals were then used to generate a correlation matrix following the methodology outlined by Richardson et al. [33,38,39] and Kadigi et al. [20,35]. A core advantage of the MVE approach is its ability to simulate multiple variables concurrently while preserving realistic correlations. This prevents implausible values, such as negative yields, and enhances the credibility of model outputs by reflecting the actual patterns observed in the data across regions and input scenarios.

To introduce correlated variability into the simulation, the derived correlation matrix was applied to transform the Independent Standard Normal Deviates (ISNDs) into Correlated Standard Normal Deviates (CSNDs). These CSNDs were subsequently converted to Correlated Uniform Standard Deviates (CUSDs) via an inverse transformation. The resulting CUSDs were used to stochastically generate empirically ranked fractional deviations, which provided the random component for simulating yield outcomes. This step was operationalized using the = CUSD() function built into the Simetar software. The MVE framework effectively captured the inherent variability in yield data while also accounting for spatial and management-related dependencies across the dataset. This allowed the simulation of spatially explicit, probabilistically valid yield scenarios that realistically reflected the diversity of farming conditions in Tanzania.

The final formulation of the MVE model is presented in Equation (4):


$$\begin{array}{c} {\tilde{y}}_{sim,i}={\bar{y}}_{c,i}*\left(\text{1}+EMP\left(S_{y,i},P\left(S_{y,i}\right),CUSD_{y,i}\right)\right) \end{array}$$ (4)

Where:

sim = Yield from the simulated dataset.

*CUSD*_*y*_ = Simetar function to simulate the correlated uniform standard deviation of random variables.

Before final analysis, the outputs generated from the MVE model were subjected to a second round of validation against historical yield data. This double-validation procedure helped ensure that the statistical properties and distributional characteristics of the simulated yields remained consistent with observed patterns. As a result, the simulated data were confirmed to be statistically reliable, well-calibrated, and suitable for evaluating the effects of fertilizer practices across Tanzania’s agroecological zones.

### Ranking of target probabilities using the stoplight function

To facilitate ranking, we consulted with experts in rice production and farmers, asking them to specify the range of food security thresholds for rice production and to set two thresholds: the maximum and minimum.

### Maximum food security threshold

The leading question here was: *What is the maximum number of 100 kg bags of rice you need to harvest per hectare to ensure your family's food is secure*? This question aims to determine the optimal level of rice production per hectare that rice farmers aspire to achieve to feel completely secure in their family’s food provision. The response helped identify the upper yield benchmark for rice that ensures sufficiency and an ideal surplus for the family’s consumption and the market, given rice's status as both a staple food and a significant agricultural commodity in Tanzania. This figure depicts the farmers’ goals under ideal growing conditions and efficient farming practices, where yields are high enough to meet all food needs and potentially generate economic benefits through sales.

### Minimum food security threshold

The leading question here was: *What is the minimum number of 100 kg bags of rice you need to harvest per hectare to ensure your family's food security*? This question seeks to establish the critical minimum yield level below which rice farmers perceive a severe risk of food insecurity. The question was designed to understand the lowest acceptable yield that would barely meet the basic food requirements of the farmer’s family without considering selling rice for income. The responses indicated the vulnerability threshold for food insecurity among smallholder rice farmers in Tanzania, highlighting the amount of rice needed to avoid the most severe economic and nutritional consequences. This threshold is crucial for planning interventions and support systems to prevent rice yields from falling below it, safeguarding against increased hunger risk and economic instability.

**Fig 1:**
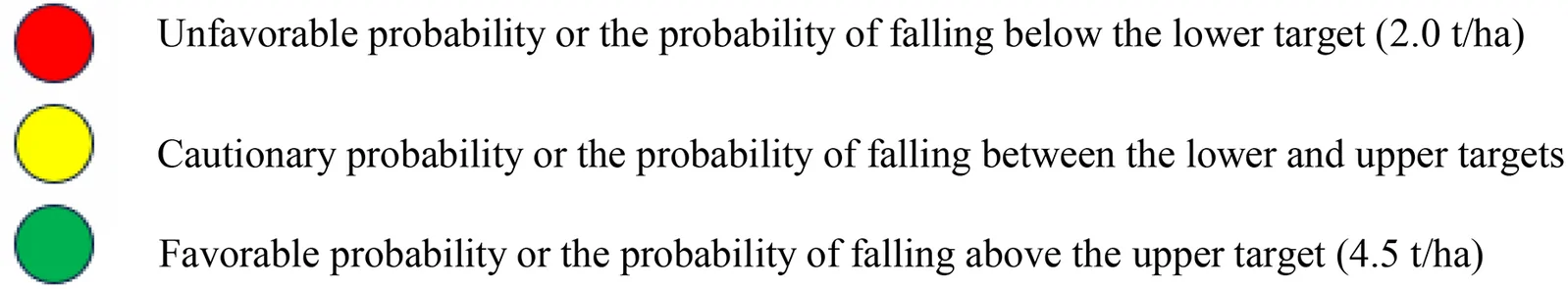
Stoplight chart for ranking of target probabilities. Red shows the unfavorable probability, yellow is cautionary probability and green is required or favorable probability.

The Stoplight Chart function was used to rank the probabilities that rice farms would achieve the maximum yield threshold and those that would fall below the minimum threshold (Fig 1). The stoplight function calculates the probabilities of (a) exceeding the upper target (*green*), (b) being less than the lower target (*red*), and (c) falling between the targets (*yellow*). The experts’ and farmers’ responses indicated that farmers were likely to be happier if their yield was equal to or greater than 4.48 t/ha; hence, the maximum threshold set was 4.5 t/ha. On the other hand, the farmers’ responses revealed that yields below 2.01 t/ha would make them unhappy, with the highest likelihood of being food-insured, so we set the minimum threshold at 2.0 t/ha.

## Results

The findings of this study were organized into four main categories. First, we validated the stochastic model used for the analysis to ensure it accurately simulated the farming practices under investigation. The results of this model validation are detailed in the supporting information section. Following validation, the second step involved examining the impact of the selected farming practices on rice farms across Tanzania, including those on the Mainland and in Zanzibar (S1 File.). Additionally, the analysis extended to assess the impact on farms located in high-producing agroecological zones, specifically the Southern Highlands Zone (SHZ) and the Eastern & Coastal zone (ECZ).

### Impact of seed and fertilizer combinations on rice yield and food security across Tanzanian farms

Table 2 presents summary statistics of rice yield (t/ha) across various farming setups in Mainland Tanzania, Zanzibar, and the entire country, highlighting differences in yield between local and improved seed use under various fertilizer regimes. On average, farms across Tanzania using local seeds without fertilizer (F1.0) recorded a yield of 2.08 t/ha, increasing slightly to 2.32 t/ha and 2.31 t/ha under organic (F1.1) and inorganic fertilizers (F1.2), respectively, suggesting that fertilizer use improves yields even with local seeds. However, yield variations exist between Zanzibar and the Mainland. For instance, Zanzibar farms under F1.0 had a lower mean yield (1.54 t/ha) than those on the Mainland (2.10 t/ha), and this trend persists across F1.1 and F1.2 setups, suggesting relatively limited productivity in Zanzibar. In contrast, farms using improved seeds (F2.0, F2.1, F2.2) showed notably higher yields. Across Tanzania, the mean yield increases from 2.08 t/ha (F2.0, no fertilizer) to 2.78 t/ha (F2.2, with inorganic fertilizer), with a peak yield of 8.65 t/ha. In Mainland Tanzania, the highest average yield was recorded under F2.2 at 3.36 t/ha, substantially outperforming yields under F1. In contrast, Zanzibar still lags behind with lower mean values (e.g., 1.76 t/ha under F2.0). The coefficient of variation (CV) indicates that variability is higher under improved seed systems without fertilizers (F2.0) and lower under inorganic fertilizer use (F2.2), particularly in Mainland Tanzania, implying more stable yield outcomes with complementary inputs. Minimum and maximum values reinforce the substantial yield potential under improved systems. These results highlight the productivity advantage of improved seeds, particularly when combined with inorganic fertilizers, and indicate regional yield disparities between the Mainland and Zanzibar. This provides strong justification for targeted interventions that promote improved seed and fertilizer adoption, particularly in low-yielding zones such as Zanzibar.

**Table 2:** Summary statistics on yield distribution (in t/ha) for farms located in Mainland Tanzania and Zanzibar.

Farms in Tanzania Zanzibar&Mainland	Summary Statistics				
	Mean	SDT	CV	Min	Max
***Farms using local seeds (plus no fertilizer, organic and inorganic fertilizers)***					
All Farms in Tanzania under_F1.0	2.08	1.13	54.58	0.13	6.23
All Farms in Tanzania under_F1.1	2.32	1.32	56.86	0.16	6.18
All Farms in Tanzania under_F1.2	2.31	1.32	54.12	0.12	6.18
All Farms in Zanzibar Tanzania under_F1.0	1.54	0.93	57.11	0.06	5.44
All Farms in Zanzibar Tanzania under_F1.1	1.64	0.84	60.38	0.04	4.94
All Farms in Zanzibar Tanzania under_F1.2	1.62	0.81	51.48	0.04	4.45
All Farms in Mainland Tanzania under_F1.0	2.10	1.14	49.72	0.13	6.23
All Farms in Mainland Tanzania under_F1.1	2.46	1.36	54.12	0.37	6.18
All Farms in Mainland Tanzania under_F1.2	2.46	1.36	55.13	0.37	6.18
***Farms using improved seeds (plus no fertilizer, organic and inorganic fertilizers)***					
All Farms in Tanzania under_F2.0	2.08	1.28	61.62	0.00	8.65
All Farms in Tanzania under_F2.1	2.48	1.34	54.05	0.62	6.18
All Farms in Tanzania under_F2.2	2.78	1.51	54.22	0.03	6.42
All Farms in Zanzibar Tanzania under_F2.0	1.76	1.42	80.76	0.02	8.65
All Farms in Zanzibar Tanzania under_F2.1	2.54	1.54	60.61	0.62	6.18
All Farms in Zanzibar Tanzania under_F2.2	2.14	1.28	59.85	0.25	6.42
All Farms in Mainland Tanzania under_F2.0	2.22	1.19	53.48	0.00	6.23
All Farms in Mainland Tanzania under_F2.1	2.41	0.98	40.86	1.16	4.94
All Farms in Mainland Tanzania under_F2.2	3.36	1.46	43.38	0.03	6.13

***Note:*** *F1.0 = farms using local seeds without fertilizers; _F1.1 farms using local seeds with organic fertilizer, and _F1.2 = farms using local seeds with organic fertilizers; _F2.0 = farms using improved seeds without fertilizers; _F2.1 farms using improved seeds with organic fertilizer; and _F2.2 = farms using improved seeds with inorganic fertilizers.*

**Fig 2:**
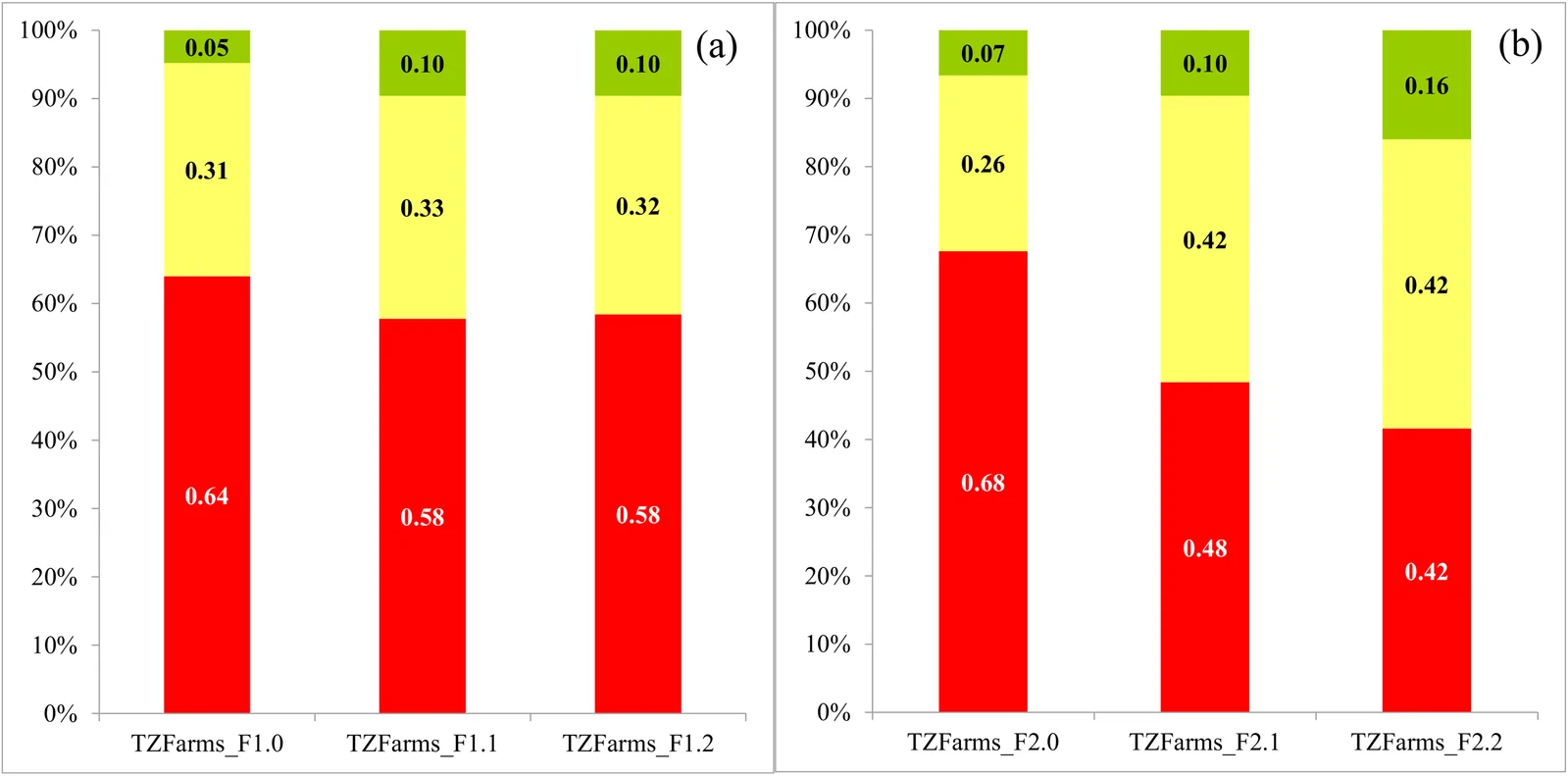
Probabilities of the productivity falling above the maximum food security threshold of 4.5 t/ha (*in green*) and less than the minimum food security threshold of 2.0 t/ha (*in red*), and the probability of falling between the two food security for rice farms using local seeds with different types of fertilizers (Figure 2a) and those farms using improved seeds (Figure 2b) for all rice farms in Tanzania. Fig 2a includes_F1.0 = Tanzanian farms using local seeds without fertilizers; _F1.1 farms using local seeds with organic fertilizer, and _F1.2 = farms using local seeds with organic fertilizers. Fig 2b includes_F2.0 = farms using improved seeds without fertilizers; _F2.1 farms using improved seeds with organic fertilizer; and _F2.2 = farms using improved seeds with inorganic fertilizers.

Fig 2 presents the impact of different seed and fertilizer combinations on rice yield probabilities across Tanzania, highlighting the significant impact of these agricultural practices on food security. The results are divided into two sets: one analyzing farms using local seeds (Fig 2a) and the other using improved seeds (Fig 2b), each with variations in fertilizer use. For farms using local seeds without any fertilizer (F1.0), the majority (64%) of yields fall below the minimum food security threshold of 2.0 t/ha, indicating high food insecurity, with only 31% achieving moderate yields and a mere 5% exceeding the maximum threshold of 4.5 t/ha. Introducing organic fertilizer (F1.1) to local seeds decreases the percentage of yields below the threshold to 58%, improves moderate yields to 33%, and increases higher yields to 10%. These findings suggest that organic fertilizers can partially alleviate the yield constraints associated with local seeds, though the improvement is modest. Switching to inorganic fertilizer (F1.2) with local seeds shows a pattern similar to that of local seeds plus organic fertilizer, with 58% of yields remaining below the threshold and almost 10% exceeding the maximum threshold. In other words, across rice farms in Tanzania, inorganic fertilizers have a relatively equal impact as organic fertilizers when local rice seeds are used.

Farmers using improved seeds with various fertilizer applications exhibit different yield probabilities. For example, farmers using improved seeds without fertilizer (F2.0) had a relatively high probability (68%) of rice productivity falling below the threshold. Still, they performed better in the high-yield (7%) category than local seeds without fertilizer (5%). Combining improved seeds with organic fertilizer (F2.1) further enhances outcomes, reducing under-threshold yields to 48% while significantly increasing moderate and high yields to 42% and 10%, respectively. This combination offers potent synergy, markedly boosting rice productivity. The most effective scenario involves improved seeds with inorganic fertilizer (F2.2), where only 42% of yields fall below the food security threshold, and an impressive 42% reach moderate yields, with 16% exceeding the high yield threshold of 4.5 t/ha. This suggests that inorganic fertilizers, when paired with improved seeds, offer the most robust solution for maximizing rice productivity and enhancing food security across Tanzanian rice farms.

### The combination of local seeds and fertilizers on rice productivity for farms located in Zanzibar and Mainland Tanzania

**Fig 3:**
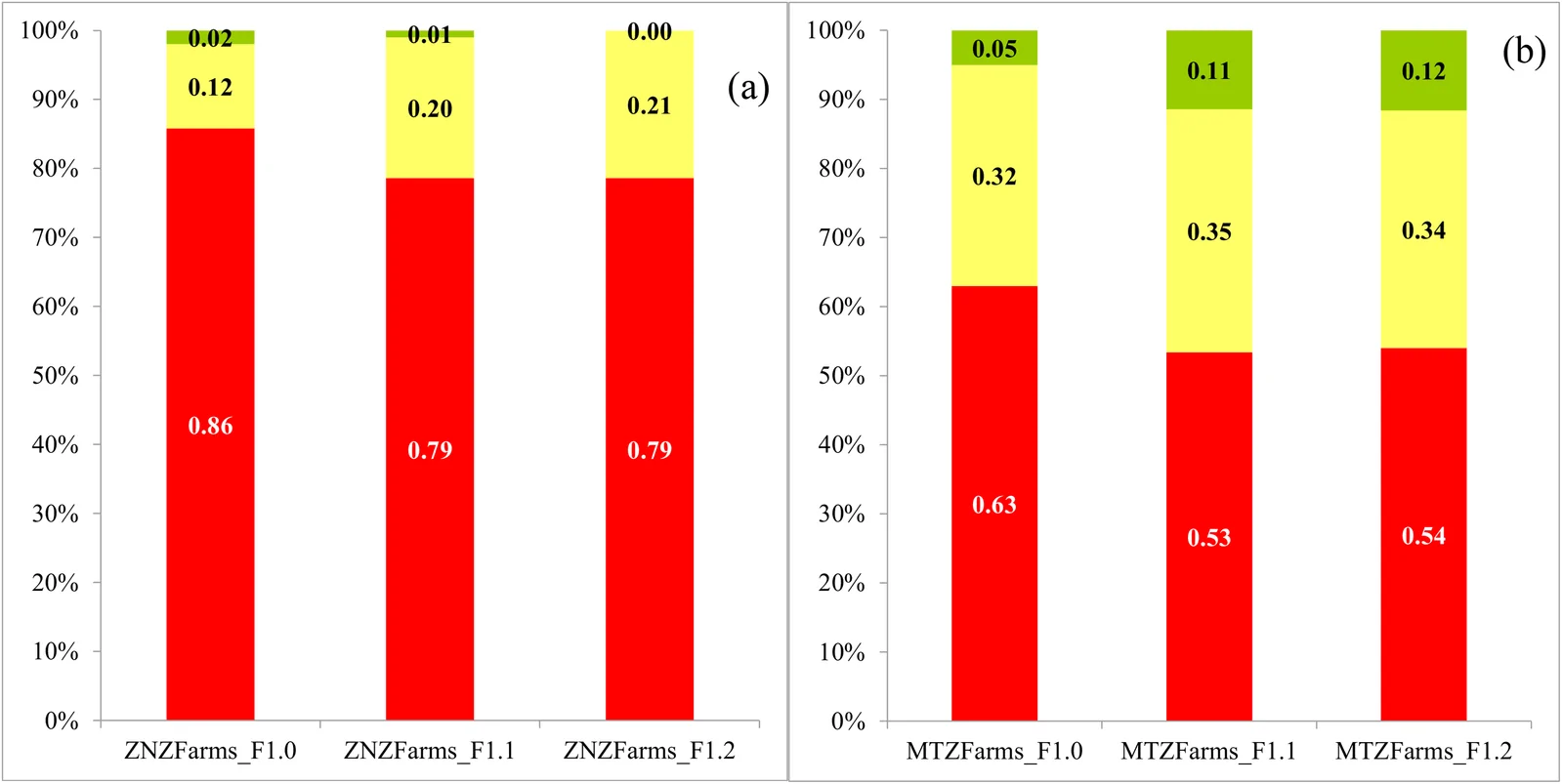
Probabilities of productivity falling above the maximum food security threshold of 4.5 t/ha (*in green*) and less than the minimum food security threshold of 2.0 t/ha (*in red*), and the probability of falling between the two food security for rice farms using local seeds with different types of fertilizers for farms located in Zanzibar (ZNZFarms, Figure 3a) and those in Mainland Tanzania (MTZFarms, Figure 3b). _F1.0 = farms using local seeds without fertilizers; _F1.1 farms using local seeds with organic fertilizer; and _F1.2 = farms using local seeds with inorganic fertilizers for both Zanzibar (Fig 3a.) and Mainland Tanzania (Fig 3b.).

Fig 3 highlights significant differences in rice productivity by fertilizer use across farms in Zanzibar and Mainland Tanzania, categorized by yield thresholds indicative of food security levels. In Zanzibar, farmers using local seeds without fertilizers exhibit a high probability (86%) of falling below the minimum food security threshold of 2.0 t/ha, indicating a severe risk of food insecurity (Fig 3a). The likelihood of achieving yields within the secure range (2.0–4.5 t/ha) is only 12%. The probability of exceeding the maximum threshold of 4.5 t/ha is extremely low at only 2%. When organic fertilizers are applied, the likelihood of achieving yields below 2.0 t/ha decreases slightly to 79%, with a corresponding increase in the likelihood of achieving food-secure yields (20%). However, the probability of exceeding 4.5 t/ha remains marginal at just 1%. Similarly, farms using inorganic fertilizers showed no significant difference from those using organic fertilizers and local seeds, indicating a virtually zero chance of surpassing the maximum food security threshold.

In Mainland Tanzania, the probability of yields falling below the minimum threshold is lower than in Zanzibar, particularly for farms using fertilizers. Rice farm families that rely solely on local seeds without fertilizers face a 63% probability of food insecurity. However, they have a slightly higher chance (32%) of achieving yields within the secure range, with 5% surpassing the maximum threshold. When organic fertilizers are introduced, the probability of food-insecure yields decreases to 53%, while 35% of farms achieve yields within the secure range, and 11% exceed 4.5 t/ha.

Similarly, using inorganic fertilizers results in a 54% probability of falling below the minimum threshold, a 34% likelihood of achieving secure yields, and a 12% chance of exceeding the maximum threshold. The results under this scenario suggest that the use of fertilizers (both organic and synthetic) plays a crucial role in improving rice productivity and reducing the risk of food insecurity among smallholder farmers in Tanzania. However, even with fertilizers, the likelihood of surpassing the maximum food security threshold remains relatively low, particularly for farms located in Zanzibar when local seeds are used.

### The combination of improved seeds and fertilizers on rice productivity for farms located in Zanzibar and Mainland Tanzania

**Fig 4:**
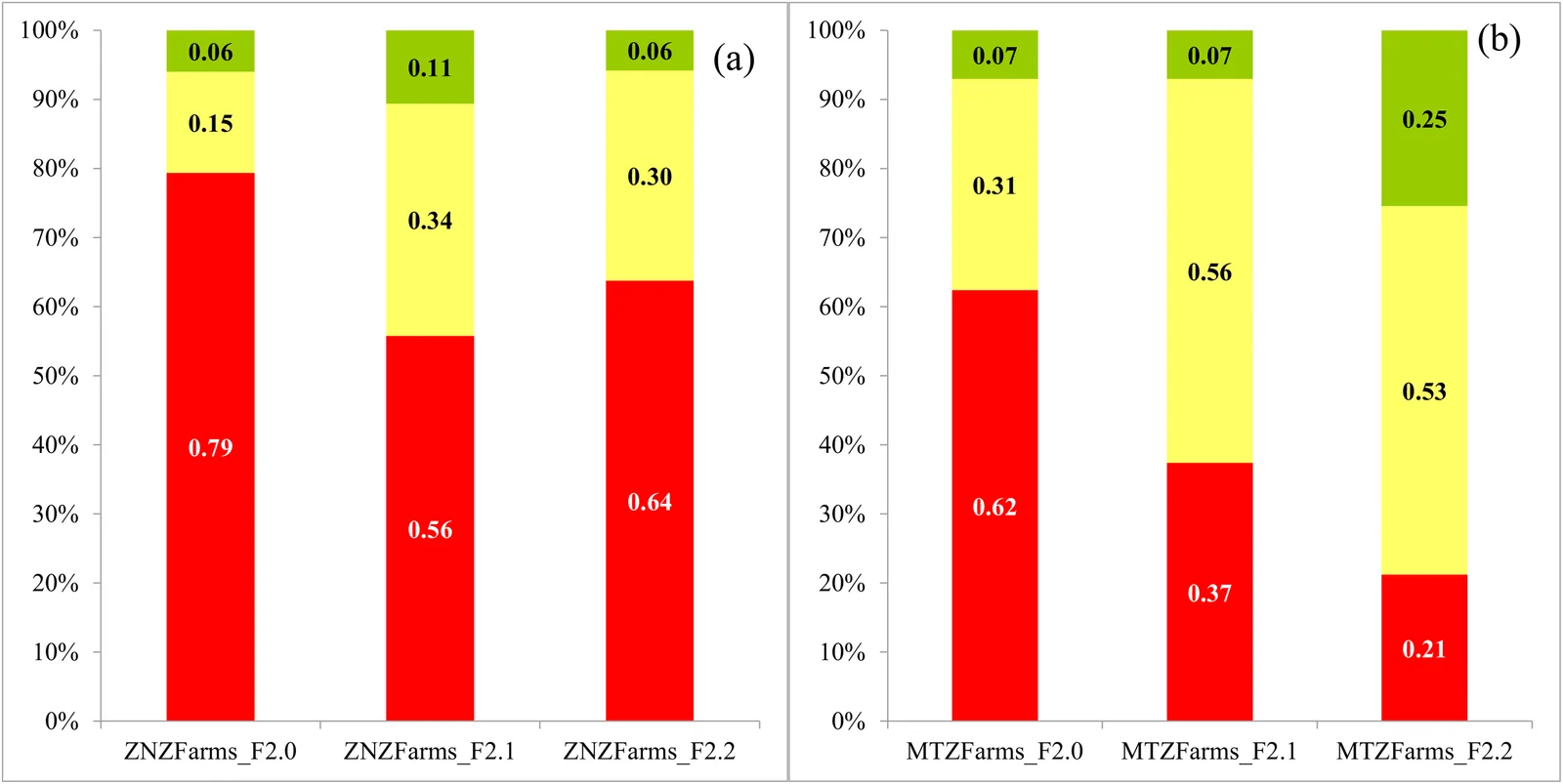
Probabilities of productivity falling above the maximum food security threshold of 4.5 t/ha (*in green*) and less than the minimum food security threshold of 2.0 t/ha (*in red*), and the probability of falling between the two food security for rice farms using improved seeds with different types of fertilizers for farms located in Zanzibar (ZNZFarms, Figure 4a) and those in Mainland Tanzania (MTZFarms, Figure 4b). _F2.0 = farms using improved seeds without fertilizers; _F2.1 farms using improved seeds with organic fertilizer; and _F2.2 = farms using improved seeds with inorganic fertilizers for both farm in Zanzibar (Fig 4a) and Mainland (Fig 4b).

Fig 4 evaluates the impact of using improved seeds with and without fertilizers on rice productivity in Zanzibar and Mainland Tanzania. In Zanzibar (Fig 4a), when using improved seeds without fertilizer (F2.0), a significant majority (79%) of yields fall below the minimum food security threshold of 2.0 tons per hectare, with only 15% reaching moderate yields between 2.0 and 4.5 tons per hectare. A mere 6% exceeding the maximum threshold of 4.5 tons per hectare. This farming practice highlights the limited effectiveness of improved seeds in enhancing productivity. Adding organic fertilizer (F2.1) improves outcomes, reducing the proportion of yields below 2.0 tons per hectare to 56%, increasing moderate yields to 34%, and slightly improving high yields to 11%. However, the introduction of inorganic fertilizer (F2.2) with improved seeds shifts the results more unfavorably, decreasing inadequate yields to 64%, with 30% achieving moderate yields and only 6% surpassing the high yield threshold, indicating a slightly lower performance compared to the use of organic fertilizers within the Island.

Meanwhile, in Mainland Tanzania (Fig 4b), improved seeds without fertilizer (FP2.0) yield results show that 62% of yields are still below the food security threshold, 31% fall within the moderate range, and 7% exceed 4.5 tons per hectare, suggesting some innate advantage of the local conditions. The results are more balanced with the incorporation of organic fertilizer (F2.1): 37% of yields remain below 2.0 tons per hectare, 56% fall within the secure range, and 7% exceed the high-yield mark, highlighting the effectiveness of organic inputs. The use of inorganic fertilizers with improved seeds (F2.2) yields the best outcomes, with only 21% falling below the threshold, 53% achieving moderate yields, and a notable 25% exceeding 4.5 tons per hectare, thereby illustrating the substantial impact of inorganic fertilizers in maximizing rice production.

### Impact of improved seeds and fertilizers on the productivity of rice farms in major producing agroecological zones of Tanzania

**Fig 5:**
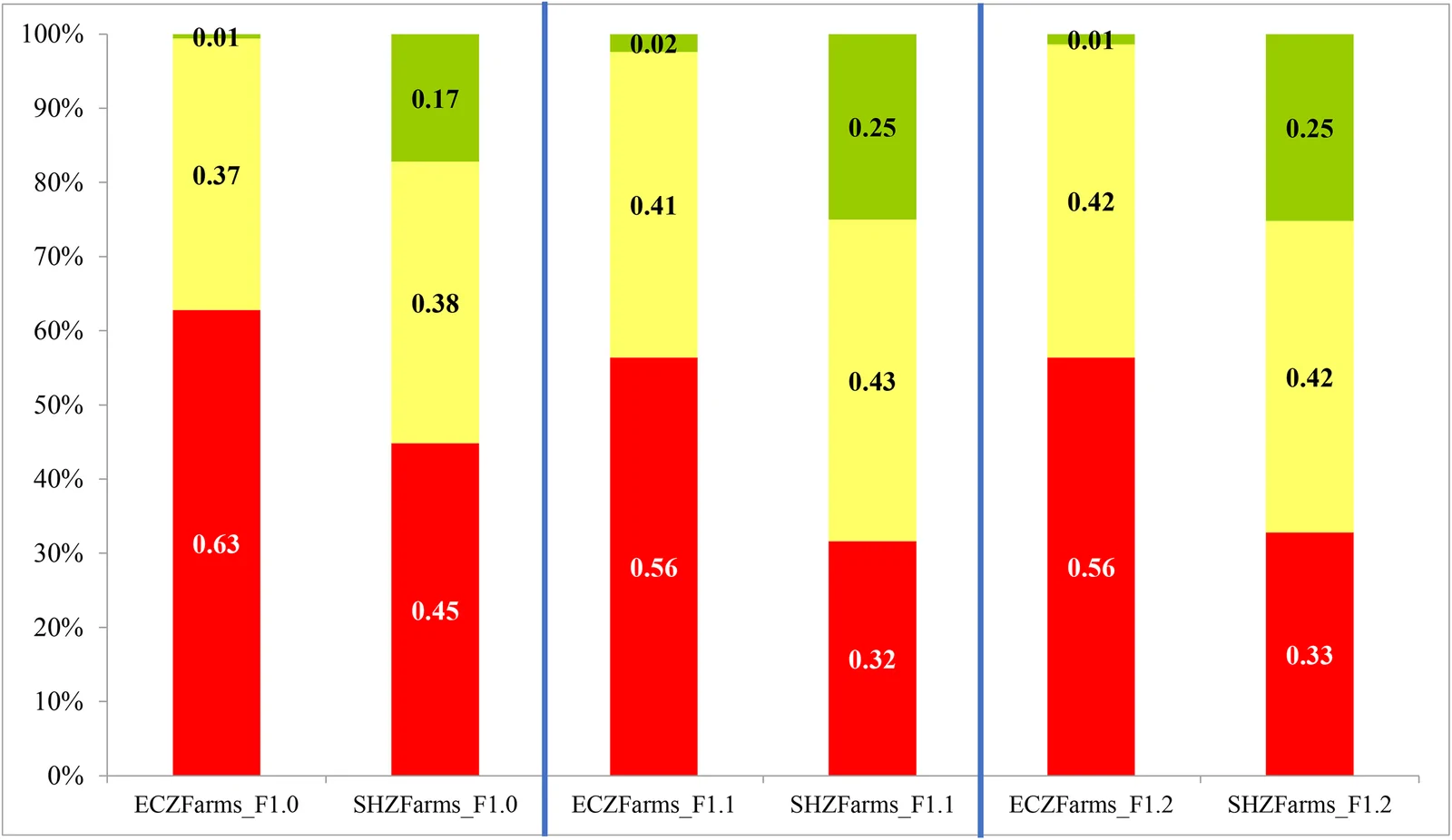
Probabilities of productivity falling above the maximum food security threshold of 4.5 t/ha (*in green*) and less than the minimum food security threshold of 2.0 t/ha (*in red*), and the probability of falling between the two food security for rice farms using local seeds with different types of fertilizers for farms in major rice producing agroecological zones (ECZ & SHZ) in Tanzania. _F1.0 = farms using local seeds without fertilizers; _F1.1 farms using local seeds with organic fertilizer; and _F1.2 = farms using local seeds with inorganic fertilizers for the selected agroecological zones.

Fig 5 displays the probabilities of rice productivity for local seed and fertilizer combinations across two major rice-producing agroecological zones in Tanzania.

For farms using local seeds without fertilizer, a significant proportion of yields fall below the minimum threshold in both zones (63% in ECZ and 45% in SHZ), indicating a high risk of food insecurity without fertilizer use, particularly in ECZ. Only a 1% probability exceeds the upper threshold in ECZ, whereas a 17% probability is observed for farms in SHZ. The yield probability falling between the minimum and maximum thresholds is 37% and 38% for farms in ECZ and SHZ, respectively, demonstrating the limited effectiveness of local seeds without supplemental nutrients. Farms using local seeds, combined with organic fertilizer, show a slight improvement in ECZ outcomes, with a notable improvement in farms located in SHZ. In ECZ, 56% of yields still fall below the food security threshold, with 41% achieving moderate yields and 2% exceeding the high yield threshold. Farms in SHZ exhibit a significant decrease (from 45% to 32%) in the probability of yield falling below the food insecurity threshold, accompanied by better performance in the high yield category (25% exceeding 4.5 t/ha), suggesting that organic fertilizers are more effective in this agroecological zone than in ECZ.

Similarly, farms using local seeds with inorganic fertilizer show a marked improvement, particularly in SHZ, where only 33% of yields fall below the threshold, 42% achieve moderate yields, and 25% exceed the high yield threshold. Adding inorganic fertilizers to farms located in ECZ has a relatively insignificant impact compared to organic fertilizers, as both scenarios still show a greater proportion (56%) of falling below the minimum threshold.

**Fig 6:**
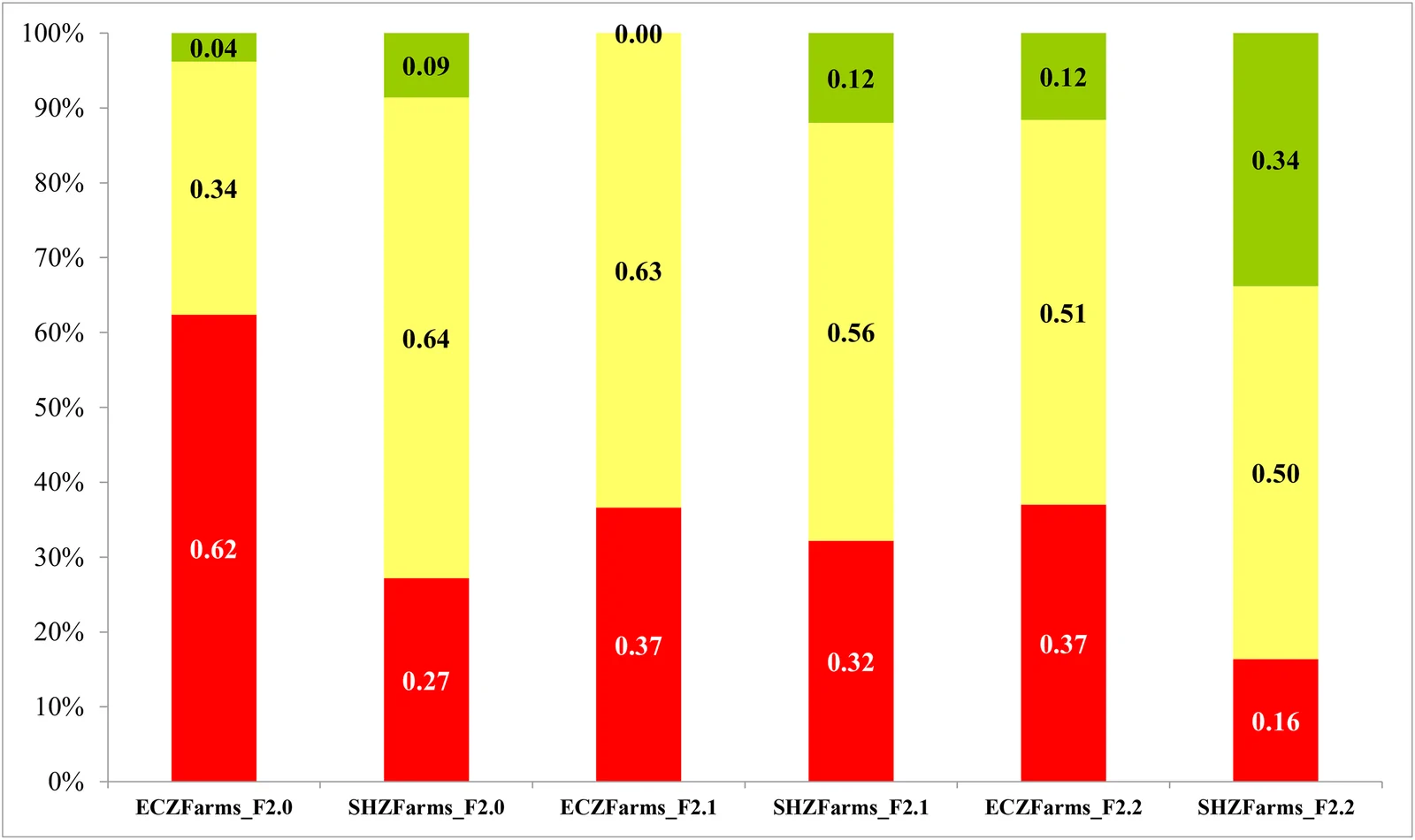
Probabilities productivity falling above the maximum food security threshold of 4.5 t/ha (*in green*) and less than minimum food security threshold of 2.0 t/ha (*in red*), and the probability of falling between the two food security for rice farms using improved seeds with different types of fertilizers for farms in major rice producing agroecological zones (ECZ & SHZ) in Tanzania. _F2.0 = farms using improved seeds without fertilizers; _F2.1 farms using improved seeds with organic fertilizer; and _F2.2 = farms using improved seeds with inorganic fertilizers for the selected agroecological zones.

Fig 6 displays the probabilities of rice productivity for improved seed and fertilizer combinations across two major rice-producing agroecological zones in Tanzania. Farms using improved seeds without fertilizer perform relatively better than those using local seeds. For example, in ECZ, 62% of yields are below the threshold, but 34% achieve moderate yields, and 4% exceed the high threshold. Although the farms in SHZ have a lower likelihood of exceeding the maximum threshold than in the above scenario, the probability of falling below the minimum value is significantly lower (27%). This indicates that improved seeds alone can improve rice productivity in SHZ.

When improved seed is combined with organic fertilizers, significant improvements are observed in both agroecological zones, particularly in achieving moderate yield levels (63% in ECZ and 56% in SHZ). This combination also results in a 37% reduction in the probability of yields falling below the minimum threshold in ECZ and a 32% reduction in SHZ, with the latter having a 12% probability of yields exceeding the maximum threshold. A similar trend was observed when improved seeds were combined with inorganic fertilizers. Still, the best outcomes are observed in this scenario, particularly in SHZ, where only 16% of yields are below the threshold, 50% achieve moderate yields, and an impressive 34% exceed the high yield threshold. ECZ also shows good performance, but with fewer yields in the highest category and a slightly higher risk of falling below the minimum threshold.

## Discussion

This analysis first examined the use of local seeds in combination with various types of fertilizers across rice farms in Tanzania, with a specific focus on differences between Mainland Tanzania and Zanzibar and the major rice-producing agroecological zones within the country. The findings reveal that the majority of yields from farms using local seeds without fertilizer fall below the critical food security threshold of 2.0 t/ha. This underscores the significant challenges farmers face when relying on traditional seed varieties without fertilizer support. The introduction of organic fertilizers led to a moderate improvement in yield. While organic fertilizers can boost soil fertility and crop yields, their impact alone may not be sufficient to increase productivity significantly [40]. Timsina [41] states that although organic fertilizers enhance soil health and prolong fertility, their nutrient release rates may not meet the immediate nutrient demands of high-yielding crop varieties.

In contrast, using inorganic fertilizers with local seeds yielded more pronounced improvements in crop yields. This strategy not only reduced the proportion of yields falling below the food security threshold but also did so more effectively than organic fertilizers. Smil [23] notes that inorganic fertilizers provide immediate nutrient availability, which is crucial for the rapid growth spurts required in intensive farming practices. Furthermore, Savary et al. [42] highlight that inorganic fertilizers can significantly improve nutrient uptake efficiency, thereby boosting crop yields more effectively than organic options in rice farming. While both types of fertilizers reduce the percentage of yields below the food security threshold, inorganic fertilizers appear slightly more effective at pushing yields into the moderate and high productivity brackets. Inorganic fertilizers offer quick results, but the sustainable use of organic fertilizers can improve soil health over the long term, thus supporting continued agricultural productivity [26,43].

As demonstrated in Mainland Tanzania, the use of inorganic fertilizers not only reduces the incidence of food insecurity but also increases the likelihood of surpassing food security thresholds, underscoring the role of fertilizers in subsistence farming and in facilitating economic gains through surplus production [9]. Robertson and Vitousek [44] noted that choosing between organic and inorganic fertilizers often involves balancing economic and ecological considerations, with cost-effective solutions that do not harm the environment being preferred. In terms of food security, enhancing fertilizer efficiency is linked to improved food security outcomes, as it directly influences yield levels and the stability of the food supply [13]. The differential impact observed between Zanzibar and Mainland Tanzania underscores the importance of regional adaptations in fertilizer application strategies [45,46].

The same analysis was also conducted in two leading rice-producing agroecological zones in Tanzania, specifically the Eastern & Coastal Zone (ECZ) and the Southern Highlands Zone (SHZ), which underlines how different fertilizers affect rice farm productivity in these zones. These results demonstrate a moderate improvement of local seeds with organic fertilizers, reflecting broader findings within agricultural research that emphasize the benefits of organic matter for improving soil structure and long-term fertility, which can lead to increased crop yields over time [27,28,47]. However, the boost in productivity may not be as immediate or significant as with inorganic fertilizers due to the slower nutrient release rates of organic inputs. Local seeds with inorganic fertilizer show substantial yield improvements in SHZ, highlighting the immediate effectiveness of inorganic fertilizers, which provide essential nutrients more directly accessible to plants. This aligns with findings from Mi et al. [48] and Iqbal et al. [29], who noted that inorganic fertilizers could rapidly address nutrient deficiencies and significantly enhance crop yields in the short term. The variability in ECZ and SHZ responses to fertilizer types underscores the influence of local soil characteristics and climatic conditions on fertilizer efficacy. Studies, such as those by Vanlauwe et al. [25], suggest that tailored fertilizer strategies that consider local environmental conditions are crucial for optimizing crop productivity. The significant reductions in the probability that yields fall below the food security threshold achieved with inorganic fertilizers, particularly in SHZ, are essential for effective food security strategies. These findings support the arguments of Pretty et al. [18], who advocate for sustainable, integrated nutrient management to increase food production.

The second analysis scenario highlighted the significant role that both improved seeds and fertilizers play in enhancing rice productivity within Zanzibar, Mainland Tanzania, and the major producing agroecological zones of Mainland Tanzania. The results are instrumental in understanding how advancements in agricultural inputs contribute to achieving higher yield thresholds, thereby impacting food security. Again, the differences in yield outcomes between Zanzibar and Mainland Tanzania can be attributed to varying soil types, climate conditions, and historical farming practices. Mainland Tanzania, with its more diverse agricultural environments, typically responds better to improved seeds and fertilizers than Zanzibar, which may have more challenging growing conditions.

The results demonstrated that in both Zanzibar and Mainland Tanzania, improved seeds alone show a notable improvement over local seeds. However, a substantial proportion of yields still fall below the food security threshold, highlighting the need for complementary inputs such as fertilizers, as Zanzibar continues to face more challenging growing conditions. Using organic fertilizers with improved seeds significantly reduces the incidence of yields below the food security threshold and increases the likelihood of achieving moderate to high yields. This finding supports the argument that organic fertilizers can be effectively integrated into rice production systems to enhance soil fertility and crop productivity [49]. However, the most pronounced improvements are seen when improved seeds are combined with inorganic fertilizers, particularly in Mainland Tanzania. This combination reduces the proportion of low-yielding outcomes and substantially increases the proportion of yields exceeding the high-productivity threshold, underscoring the efficacy of inorganic fertilizers in supplying the nutrients required for optimal plant growth [24].

Johnson et al. [15], in their study on Inorganic fertilizer use and its association with rice yield gaps in sub-Saharan Africa, found that while organic fertilizers improve soil health over the long term, inorganic fertilizers often offer immediate improvements in yield due to their quick nutrient release, supporting the trends observed in our findings. Tsujimoto et al. [24] discuss the genetic potential of improved rice varieties to optimize nutrient uptake, which is critical for realizing the full benefits of inorganic fertilizers, as evidenced by higher yields when these inputs are combined. Reicosky et al. [50] emphasize the importance of tailoring rice farming techniques to specific agroecological zones, noting that both the type of seed and fertilizer, as well as their interaction with the local environment, significantly influence productivity. FAO [9] reports emphasize the need to integrate both types of fertilizers to balance immediate yield needs and long-term sustainability, corroborating the varied responses observed across different treatments in the figures. On the other hand, the World Bank provides insights into agricultural policy development, suggesting that policies that support access to and use of improved seeds and organic and inorganic fertilizers can enhance food security in regions like Tanzania [10].

Results from improved seeds combined with organic and inorganic fertilizers across major rice-producing zones in Tanzania reveal that improved seeds alone yield notable benefits in reducing the percentage of yields below the food security threshold, particularly in the Southern Highlands Zone (SHZ). This aligns with research by Tsubo et al. [51], who found that improved seeds could significantly enhance crop resilience and yield, even under less-than-optimal conditions. The substantial improvement in yield probabilities with the use of organic fertilizers, especially in achieving moderate to high yields, demonstrates the value of integrating organic inputs with improved seed varieties. This is supported by Scoones [52], who argues that organic fertilizers supply essential nutrients and enhance soil health, both of which are crucial for sustainable agricultural productivity.

When inorganic fertilizers are used in conjunction with improved seeds, they yield the best results across both zones, particularly in the SHZ. This finding is consistent with studies by Snapp et al. [30], who note that inorganic fertilizers can quickly correct nutrient deficiencies, leading to rapid, significant increases in crop yields. The variations in response between the Eastern & Coastal Zone (ECZ) and SHZ highlight the influence of local environmental factors on the effectiveness of fertilizers and seeds. Tailoring fertilizer and seed strategies to specific agroecological conditions is crucial, as demonstrated by Vanlauwe et al. [25], underscoring the need for context-specific agricultural innovations.

The findings of this study reinforce the view that improved seeds and fertilizer use are effective climate adaptation strategies in Tanzania’s rain-fed rice production systems. Climate adaptation in agriculture is fundamentally concerned with reducing vulnerability to climate variability, stabilizing production under uncertainty, and strengthening household resilience. By focusing on yield distributions and threshold probabilities rather than on mean outcomes alone, this study shows that improved seeds, particularly when combined with inorganic fertilizers, significantly reduce the probability that yields fall below the minimum food-security threshold while increasing the likelihood of achieving higher yield targets. This reduction in downside risk is especially critical in the context of increasing rainfall variability and temperature stress affecting Tanzanian agriculture [2,11,31]. The observed heterogeneity between Mainland Tanzania and Zanzibar further underscores that climate adaptation is context-specific, requiring location-tailored input strategies and complementary interventions to fully realize productivity and resilience gains [16,25].

These results also align closely with the United Nations Sustainable Development Goals (SDGs), particularly SDG 2 (Zero Hunger), SDG 1 (No Poverty), and SDG 13 (Climate Action). By reducing the probability of food-insecure yield outcomes, improved seed–fertilizer combinations directly support SDG 2 by enhancing household food availability and national food supply stability [9,13]. At the same time, higher probabilities of achieving moderate-to-high yields increase the potential for marketable surplus and income generation, contributing to poverty reduction under SDG 1. From a climate perspective, the ability of these input strategies to reduce yield risk and stabilize production under variable climatic conditions strengthens adaptive capacity, consistent with SDG 13 [32]. Taken together, the probabilistic evidence presented in this study underscores that improved seeds and fertilizers are not only productivity-enhancing inputs but also key components of climate-resilient and food-security-oriented agricultural development pathways in Tanzania.

## Conclusions

A comprehensive analysis of rice productivity across rice farms in Zanzibar and Mainland Tanzania, using various combinations of seeds and fertilizers, has elucidated several crucial insights and strategic implications for enhancing the country's agricultural output and food security. These findings underscore the pivotal roles of both seed type (local and improved) and fertilizer choice (organic and inorganic) in determining yield outcomes and their subsequent impact on food security. The study consistently highlights the significant impact of fertilizers on improving rice yields. In particular, inorganic fertilizers can enhance yields more effectively than organic alternatives across various agroecological zones. This is likely due to their rapid nutrient release, which meets the immediate nutrient needs of growing rice plants. Improved seeds alone have demonstrated the capacity to increase productivity, even without the addition of fertilizers. However, their potential is maximized when combined with inorganic fertilizers, yielding the highest yields and surpassing critical food security thresholds.

While organic fertilizers contribute positively to long-term soil health and sustainability, they are generally less effective at immediately boosting yields compared to inorganic fertilizers. However, combining organic fertilizers with improved seeds shows promising results, particularly in Tanzania's southern highlands, suggesting that a hybrid approach might be beneficial under certain conditions. The variation in fertilizer performance between Zanzibar and Mainland Tanzania highlights the necessity for region-specific agricultural practices. Tailored strategies that consider local soil conditions, climate, and crop needs are essential for optimizing fertilizer efficacy and overall crop productivity. The findings advocate for policy interventions supporting access to improved seeds and inorganic fertilizers. Additionally, educational programs for farmers on how to effectively use these inputs could further enhance productivity. Long-term sustainability can be encouraged through integrated approaches that combine organic and inorganic fertilizers, balancing immediate yield needs with environmental sustainability.

From the analysis, the study recommends the following:

i Promoting the use of scientifically developed seeds and readily available inorganic fertilizers can significantly improve yield outcomes.
ii Continued research into crop-specific fertilizer formulations and ongoing support through extension services help tailor farming practices to regional conditions, maximizing the benefits of agricultural inputs.
iii Encourage the integration of organic fertilizers into conventional farming systems to enhance soil health and sustainability, thereby ensuring long-term agricultural productivity.
iv Develop and implement policies that subsidize the cost of critical inputs, such as improved seeds and inorganic fertilizers, making them more accessible to smallholder farmers.

This analysis confirms that strategic enhancements in seed and fertilizer use can profoundly influence rice productivity and food security in Tanzania. Tanzania can substantially improve agricultural productivity and sustainability by adopting an integrated approach that combines the immediate effectiveness of inorganic fertilizers with the resilience of improved seeds and the long-term benefits of organic fertilizers. These strategies address immediate food security concerns and contribute to the sustainable intensification of agriculture, which is essential for the household and the nation’s economic and environmental future.

Lastly, this study does not model the socioeconomic and institutional determinants of farmers’ seed and fertilizer choices (e.g., access to credit and extension services, market distance, risk preferences, gender, or education). Future work could integrate adoption models (e.g., logit/probit/multinomial choice) with the probabilistic yield-risk framework used here to jointly explain why farmers choose particular input bundles and how those bundles translate into food-security outcomes under uncertainty. Future research could also integrate NSCA crop data with specialized livestock survey datasets to explicitly analyze mixed crop–livestock systems and assess how livestock ownership interacts with seed and fertilizer use to influence yield risk and food-security outcomes.

## Supporting information

S1 File S1. Stochastic model validation; S1 Fig. (DOCX)

S2 File Minimum dataset used for this study. The minimum dataset used for this study is attached as: S2_File.xlsx.
(XLSX)
