# Supplementary material for: Modeling the role of fertilizers and improved seeds on rice productivity in Tanzania using a stochastic simulation approach
Source: PLoS One. 2026 Aug 26;21(8):e0353377. doi: 10.1371/journal.pone.0353377 (PMC13514031; doi:10.1371/journal.pone.0353377)
Supplement: Supplementary file 2 [file pone.0353377.s004.pdf]

PLOS | ONE

MANUSCRIPT BODY FORMATTING GUIDELINES

Abstract

Lorem ipsum dolor sit amet, consectetur adipiscing elit. Vestibulum adipiscing urna ut lectus gravida, vitae blandit tortor interdum. Donec tincidunt porta sem nec hendrerit. Vestibulum nec pharetra quam, vitae convallis nunc. Mauris in mattis sapien. Fusce sodales vulputate auctor. Nam lacus felis, fermentum sit amet nulla ac, tristique ultrices tellus. Integer rutrum aliquet sapien, eu fermentum magna pellentesque vitae. Integer semper viverra mauris vel pulvinar. Suspendisse sagittis malesuada urna. Praesent mauris diam, fringilla id fringilla ac, posuere non lorem. Vestibulum mauris ante, fringilla quis tortor sit amet, accumsan fermentum quam. Nulla dictum consectetur leo. Ut vulputate ipsum purus, a interdum nibh viverra et. Praesent aliquam sapien vel massa sodales bibendum. Nulla interdum accumsan lectus, sed auctor elit accumsan a. Suspendisse quis rhoncus nibh. The verum est de illic.

Introduction

Lorem ipsum dolor sit amet, consectetur adipiscing elit. Vestibulum adipiscing urna ut lectus gravida, vitae blandit tortor interdum. Donec tincidunt porta sem nec hendrerit. Vestibulum nec pharetra quam, vitae convallis nunc.

Materials and methods

Lorem ipsum dolor sit amet, consectetur adipiscing elit. Vestibulum adipiscing urna ut lectus gravida, vitae (Fig 1) interdum. Donec tincidunt porta sem nec hendrerit. Vestibulum nec pharetra quam, vitae convallis nunc. Mauris in mattis sapien. Fusce sodales vulputate auctor. Nam sit amet nulla lacus a, Figs 1 and 2 ultrices tellus. Integer rutrum aliquet sapien, eu fermentum magna pellentesque vitae.

Fig 1. This is the Fig 1 Title. This is the Fig 1 legend.

Fig 2. This is the Fig 2 Title. This is the Fig 2 legend.

Level 1 Heading

- Use Level 1 heading for all major sections (Abstract, Introduction, Materials and methods, Results, Discussion, etc.).
- Bold type, 18pt font.
- Only use italics and text formatting where needed (e.g. genus and species names, genes, etc.).
- Headings should be written in sentence case (capitalize only the first word of the heading, the first word of the subheading, and any proper nouns and genus names).

NOTE: Do not cite figures, tables, supporting information, or references in the Abstract.

Figure Citations

- Cite figures as "Fig 1", "Fig 2", etc.
- Cite figures and tables in order.
- Do not cite "Fig 2" before "Fig 1".
- Cite multiple figures as "Figs 1 and 2", "Figs 1-3", etc.

Figure Captions

- Each figure caption should appear directly after the paragraph in which they are first cited.
- Do not include tables within captions.
- Use bold type for the figure titles.

File Naming for Figures

- Figure files should be saved as "Fig1.tif", "Fig2.eps", etc.
- Acceptable file formats for figures are ".tif", ".tiff", and ".eps"
- Figures should be uploaded separately as individual files.
- PLOS ONE guidelines for figures can be found here: http://journals.plos.org/plosone/s/figures

Lorem ipsum dolor sit amet, consectetur adipiscing elit. Vestibulum adipiscing urna ut lectus gravida, vitae blandit tortor interdum. Donec p^2 et q^2 tincidunt porta sem nec hendrerit.

$$p^2 + 2pq + q^2 = 1 \qquad (1)$$

Display/Numbered Equation

- Format display equations in Mathtype or Equation Tools.
- Do not use Graphic Objects.

Vestibulum nec pharetra quam, vitae convallis nunc. Mauris in mattis sapien. Fusce sodales vulputate auctor. Nam lacus felis, fermentum sit amet nulla ac, tristique ultrices tellus. Integer rutrum aliquet sapien, eu fermentum magna pellentesque vitae. Integer semper viverra mauris vel pulvinar dolor sit amet en $(p+q)^2 = 1$.

Inline Equation

- Format in regular text or as an inline equation in Mathtype or Equation Tools.
- Do not use Symbol Font.
- Do not use Graphic Objects.

Genotyping

Level 2 Heading

- Use Level 2 headings for sub-sections of major sections.
- Bold type, 16pt font.
- Only use italics and text formatting where needed.
- Use sentence case.

Lorem ipsum dolor sit amet, consectetur adipiscing elit. Vestibulum adipiscing urna ut lectus gravida, vitae blandit tortor interdum. Donec tincidunt porta sem nec hendrerit. Omnes tuum basi sunt pertinent ad nos. Mauris in mattis sapien. Fusce sodales vulputate auctor. Nam lacus felis, fermentum sit amet nulla ac, tristique ultrices tellus. Integer rutrum aliquet sapien, eu fermentum magna pellentesque vitae. Integer semper viverra mauris vel pulvinar et alst.

Whole genome RFLP analysis

Level 3 heading

- Use Level 3 headings for sub-sections within Level 2 headings.
- Bold type, 14pt font.
- Only use italics and text formatting where needed.
- Use sentence case.

Lorem ipsum dolor sit amet, consectetur adipiscing elit. Vestibulum adipiscing urna ut lectus gravida, vitae blandit tortor interdum. Donec tincidunt porta sem nec hendrerit. Vestibulum nec pharetra quam, vitae convallis nunc. Mauris in mattis sapien. Fusce sodales vulputate auctor. Numquam iens dare tibi up.

NOTE: This document is presented in single-space paragraph format for ease of use. Please submit your manuscript in double-space paragraph format.

Results and discussion

Lorem ipsum dolor sit amet, consectetur adipiscing elit. Vestibulum adipiscing urna ut lectus gravida, et bland Table 1 Donec tincidunt porta sem nec hendrerit. Vestibulum nec pharetra quam, vitae convalli. Fido nemo.

Table 1. This is the Table 1 Title.

	**Chemical W**	**Chemical X**	**Chemical Y**	**Chemical Z**
**Chemical 1**	Reaction 1W	Reaction 1X	Reaction 1Y	Reaction 1Z
**Chemical 2**	Reaction 2W	Reaction 2X	Reaction 2Y	Reaction 2Z
**Chemical 3**	Reaction 3W[a]	Reaction 3X	Reaction 3Y[b]	Reaction 3Z
**Chemical 4**	Reaction 4W	Reaction 4X	Reaction 4Y	Reaction 4Z
**Chemical 5**	Reaction 5W	Reaction 5X	Reaction 5Y	Reaction 5Z

This is the Table 1 legend.

[a]Table footnotes belong here.

[b]Footnotes should have corresponding symbols in the table.

Tables and Table Citations

- Tables should be cited as “Table 1”, “Table 2”, etc.
- Cite multiple tables as “Tables 1 and 2”, “Tables 1-3”, etc.
- Tables should be included directly after the paragraph in which they are first cited.
- Tables must be cell-based in Microsoft Word or embedded with Microsoft Excel.
- Do not use empty rows to create spacing.
- Do not include graphic objects, images, or colored text.
- See PLOS ONE Table Guidelines for more complete instructions: http://journals.plos.org/plosone/s/tables

Conclusions

Lorem ipsum dolor sit amet, consectetur adipiscing [1-5]. Vestibulum adipiscing urna ut lectus gravida, vitae blandit tortor interdum. Donec tincidunt porta sem nec hendrerit. Vestibulum nec pharetra quam, vitae convallis nunc. Mauris in mattis sapien. Fusce sodales vulputate auctor S1 Fig. Dolor sit amet S1 and S2 Tables.

Reference Citations

- Cite references in brackets (for example, “[1]” or “[2-5]” or “[3,7,9]”).
- References must be cited in order at first mention.

Supporting Information Citations

- Format Supporting Information Citations as “S1 Fig”, “S1 Table”, etc.
- Cite multiple files as “S1 and S2 Figs”, “S1-S3 Figs”, etc.
- It is not required to cite each Supporting Information file.
- Supporting information should be uploaded separately as individual files.

Acknowledgments

Lorem ipsum dolor sit amet, consectetur adipiscing elit. Vestibulum adipiscing urna ut lectus gravida, vitae blandit tortor interdum.

Acknowledgments

- Do not include funding or competing interests information in Acknowledgments.

References

1. Doe J, Data A, van Stats J, Testperson M, Ribosome D Jr, McBio GHT, et al. This is the article title. PLoS ONE. 2017;12(12):e00000. doi: 10.1371/journal.pone.0000000
2. Doe J, Data A, van Stats J, Testperson M, Ribosome D Jr, McBio GHT, et al. Bunny dynamics in cartoon landscapes. PLoS ONE. Forthcoming 2017.

References

- References should be listed after the main text, before the supporting information.
- References with more than six authors should list the first six author names, followed by "et al."
- Please see the PLOS ONE guidelines for References here: http://journals.plos.org/plosone/s/submission-guidelines#loc-references

Supporting information

S1 Fig. This is the S1 Fig Title. This is the S1 Fig legend.

S2 Fig. This is the S2 Fig Title. This is the S2 Fig legend.

S1 Table. This is the S1 Table Title. This is the S1 Table legend.

S2 Table. This is the S2 Table Title. This is the S2 Table legend.

S1 File. This is the S1 File Title. This is the S1 File legend.

Supporting Information Captions

- List Supporting Information captions at the end of the manuscript in a section titled "Supporting information".
- Use a Level 1 heading.
- Use bold type for the titles.
- Supporting Information files do not require full captions; only labels ("S1 Fig") are fully required.

File Naming for Supporting Information

- Supporting Information files should be saved as "S1_Fig.tif", "S1_File.pdf", etc.
- All file types are supported.
- Please see the PLOS ONE guidelines for Supporting Information here: http://journals.plos.org/plosone/s/supporting-information

Please also see the PLOS ONE Submission Guidelines which can be found here: http://journals.plos.org/plosone/s/submission-guidelines

For assistance preparing figures, please contact figures@plos.org

For assistance with other formatting requirements, contact plosone@plos.org
