# Supplementary material for: Modeling the role of fertilizers and improved seeds on rice productivity in Tanzania using a stochastic simulation approach
Source: PLoS One. 2026 Aug 26;21(8):e0353377. doi: 10.1371/journal.pone.0353377 (PMC13514031; doi:10.1371/journal.pone.0353377)
Supplement: Supplementary file 3 [file pone.0353377.s001.docx]

**S1. Stochastic model validation**

The validation of the stochastic model was conducted using probability density function (PDF) charts to compare observed and simulated yield distributions. These comparisons were made across various farming scenarios in Tanzania, including:

i. Farms using local seeds without fertilizer (F1.0)
ii. Farms using local seeds with organic fertilizers (F1.1)
iii. Farms using local seeds with inorganic fertilizers (F1.2)
iv. Farms using improved seeds without fertilizer (F2.0)
v. Farms using improved seeds with organic fertilizers (F2.1)
vi. Farms using improved seeds with inorganic fertilizers (F2.2)

The PDF charts in S1 Fig show a striking resemblance between the observed and simulated distributions, suggesting a strong correlation. This visual alignment provides compelling evidence that the simulation model accurately captures the underlying statistical properties of the real-world farming practices under study. The close match between the observed and simulated data underscores the model’s reliability and its ability to replicate actual conditions and outcomes in the agricultural context.

**S1 Fig.** PDF charts show the comparison of observed and simulated distributions for a yield of all farms in Tanzania using local seeds without fertilizer (F1.0), farms using local seeds + organic fertilizers (F1.1), farms using local seeds + inorganic fertilizers (F1.2), farms using improved seeds without fertilizer (F2.0), farms using improved seeds + organic fertilizers (F2.1), farms using improved seeds + inorganic fertilizers (F2.2).
