# Supplementary material for: Modeling the role of fertilizers and improved seeds on rice productivity in Tanzania using a stochastic simulation approach
Source: PLoS One. 2026 Aug 26;21(8):e0353377. doi: 10.1371/journal.pone.0353377 (PMC13514031; doi:10.1371/journal.pone.0353377)
Supplement: Supplementary file 4 [file pone.0353377.s006.docx]

**Response to editor’s raised two issues**

May 15, 2026

PONE-D-25-39698R1

Modeling the role of fertilizers and improved seeds on rice productivity in Tanzania using a stochastic simulation approach

Dear Dr. Kadigi,

We've checked your submission and before we can proceed, we need you to address the following issues:

1. When completing the data availability statement of the submission form, you indicated that you will make your data available on acceptance. We strongly recommend all authors decide on a data sharing plan before acceptance, as the process can be lengthy and hold up publication timelines. Please note that, though access restrictions are acceptable now, your entire data will need to be made freely accessible if your manuscript is accepted for publication. This policy applies to all data except where public deposition would breach compliance with the protocol approved by your research ethics board. If you are unable to adhere to our open data policy, please kindly revise your statement to explain your reasoning and we will seek the editor's input on an exemption. Please be assured that, once you have provided your new statement, the assessment of your exemption will not hold up the peer review process.

**Response:** We thank the Editor for highlighting this important issue regarding the Data Availability Statement. In response, we have revised and clarified the statement in the submission system. Specifically, we clarify that the study uses secondary data obtained from the 2019/20 National Sample Census of Agriculture (NSCA), conducted by the Tanzania National Bureau of Statistics (NBS). These data are publicly accessible upon formal request and approval from the NBS under their data access policy.

In addition, all summary statistics, analytical outputs, simulation procedures, and supplementary materials necessary to reproduce the findings have been provided within the manuscript and/or Supplementary Materials. We remain fully committed to complying with the journal’s open data policy and will ensure that all permissible materials required for reproducibility are made accessible upon acceptance of the manuscript.

2. We note that your author list was updated during the revision process. In order to add or remove authors or update the order of the author byline after initial submission, we ask that authors complete an Authorship Change Request form. You may review our full authorship change policy and download the Authorship Change Request form here: <https://journals.plos.org/plosone/s/authorship#loc-authorship-changes>.

Please fill out all required sections of the form. If you are adding or removing more than 2 authors, you can complete multiple forms and submit as many forms as needed to reflect the updates. Please return the form(s) as an attachment by emailing [plosone@plos.org](mailto:plosone@plos.org) or by uploading it as a submission file labeled with the file type ‘Other’. The form will be reviewed by PLOS staff for approval. Should the changes be approved, we will require written confirmation from all authors confirming that they approve of the changes. Please note that if your manuscript is accepted, we will not be able to complete the publication process without the completed form.

**Response:** We thank the Editorial Office for bringing this matter to our attention. During the revision process, there was a temporary adjustment to the author list following internal discussions about contribution levels. However, to avoid unnecessary administrative procedures and maintain consistency with the original submission, we have decided to retain the authorship list from the original submission, including Dr. Lazaro Kagata and Prof. Stefan Sieber, in the revised manuscript. Accordingly, the authorship order and composition have now been restored to match the original submission, and no further authorship changes are being requested. We therefore believe that the Authorship Change Request form will no longer be necessary. We sincerely apologize for any inconvenience or confusion this may have caused and thank the Editorial Office for the guidance provided.

We've returned your manuscript to your account. Please resolve these issues and resubmit your manuscript within 21 days. If you need more time, please email the journal office at [plosone@plos.org](mailto:plosone@plos.org). We are happy to grant extensions of up to one month past this due date. If we do not hear from you within 21 days, we will withdraw your manuscript.

Please log on to PLOS Editorial Manager at <https://www.editorialmanager.com/pone/> to access your manuscript. You will find your manuscript in the 'Submissions Sent Back to Author' link under the New Submissions menu. Be sure to remove your previous manuscript file if you are uploading a new file in response to these requests. After you've made the changes requested above, please be sure to view and approve the revised PDF after rebuilding the PDF to complete the resubmission process.

We are requesting these changes to comply with the PLOS ONE submission guidelines (<https://journals.plos.org/plosone/s/submission-guidelines>). Please note that we won't send your manuscript for review until you have resolved the above requests.

Thank you for submitting your work to PLOS ONE and supporting our mission of Open Science.


Kind regards,

Maidelyn R. Peregrin

PLOS One
