# Supplementary material for: Modeling the role of fertilizers and improved seeds on rice productivity in Tanzania using a stochastic simulation approach
Source: PLoS One. 2026 Aug 26;21(8):e0353377. doi: 10.1371/journal.pone.0353377 (PMC13514031; doi:10.1371/journal.pone.0353377)
Supplement: Peer review history

# Peer review: Decision Letter 0

**DOI:** 10.1371/journal.pone.0353377.r001

**Authors:** Nickson Erick Otieno

24 Nov 2025

PONE-D-25-39698

Modeling the Role of Fertilizers and Improved Seeds on Rice Productivity in Tanzania using a Stochastic Simulation Approach

PLOS ONE

Dear Dr. Kadigi,

Thank you for submitting your manuscript to PLOS ONE. After careful consideration, we feel that it has merit but does not fully meet PLOS ONE’s publication criteria as it currently stands. Therefore, we invite you to submit a revised version of the manuscript that addresses the points raised during the review process.

==============================

The two reviewers both agree about the importance of the study, and its potential contribution to knowledge about the potential significance of improved rice production for local and national food security in a changing climate

However, they (and especially reviewer #1) feel that there are a number of technical inadequacies that undermine the quality of the paper, making it unsuitable for publication in its current state.

The study appears to have been pegged or relied almost entirely on analyses of rice production choice pathways (seed and fertilizer type) datasets using stochastic simulation (SS), reportedly anchored on the works of one Richardson. Although the analysis procedure is fairly well outlined, it was not very clear why the authors centered primarily on this one analytical technique and ignored all other standard strategies for assessing sociological data, which would have served to greatly complement the SS element and thus provide a more complete picture of the relative merits of fertilizer and seed choice by farmers.

The Introduction does not clearly articulate through literature review, what gap the research fills in knowledge on the subject, or how it advances existing knowledge based on past studies. This should entail reviewing literature up to date. The literature review should also include some introductory material on the technique adopted by the authors in their study – stochastic simulation. For instance, what are the relative merits of probabilistic/stochastic modeling for this study in comparison to other alternative analytical approaches? Furthermore, the introduction contains no literature review at all, on the relative merits of the choice or types of fertilizer and seeds, and how this relates to rural and national rice production goals/scenarios. There are tons of existing literature on that.

For such an elaborate attempt at statistical modeling, there must be at least 2 hypotheses to be tested. Not only were there no hypotheses stated or tested, there was no clear list of objectives underpinn9ng the investigation. The introduction must end with a clear list of objectives, and these should form the basis for the set of testable hypotheses for which the stochastic simulation will be run. Further down into Discussion, include in some greater detail how you findings align with the climate adaptation concept (which you mention) within the context of United Nations sustainable development goals relevant to the subject matter

In methods, this study primarily involved interviews with farmers, but this element is not mentioned anywhere in the paper. Instead, all focus seems to have been given to the aspect of simulation. Stochastic simulation may have been suitable for your purpose in integrating many elements of farm management, but why were other standard procedures for assessing sociological datasets (there are numerous ones) not included to complement or supplement simulation models?

There is no mention of how many households were sampled with the various regional Primary sampling units (PSU). Clear indication of this sample size and how it was incorporated in the models (what term in your stochastic simulation formula represents it?).

The terms you use such as ‘proportionately distributed’ and ‘systematically selected’ what do they mean in reference to sampling design and representativeness?

As a matter of fact, were all rice growers who also reared livestock included in the dataset, or some sort of sampling criteria used to pick a small sample in each place. Tis information and the ultimate sample size per PSu need to be provided

Also needed is information as to how up-to-date the census information was (for instance, a decades-old census dataset might be less useful currently). Include also the censusing authority, whether government or other agency, with citation of the census relevant actual census report

In many cases within Methods and analyses or results, the authors merely refer readers to various references especially Richardson. Methods must be described real-time for readers to be able to relate the study protocol to the study title, objectives, analyses and results reported, as well as are able to reproduce the procedure. They must not be asked to fish out the methods descriptions for themselves from another publication. In particular, each of the terms of the formula presented must be fully defined and described as well as justified for its value for the study’s objectives.

For instance, a term multivariate empirical distribution is abruptly mentioned as n approach for integrating various variables in simulation modeling. But no details are given, the readers being asked to find out from Richardson, 2000.  NO other mention is made of the term, and there is no indication as to how or where exactly it was indeed applied.

How datasets were pre-treated before simulation should be clearly described, especially as there were not only two variables (rice seed type and fertilizer type) but also spatial units (regions/agroecological zones); the element of crop vs. livestock production, including how these were standardized or re-scaled from the various components of measurement units. It is unlikely that any simulation model function would simply accommodate any number of data elements regardless of scale or unit.

Information presented in section 2.3 should best be presented in form of a table

At the end of the Discussion or at Conclusion, there must subsequently then be a statement outlining the extent to which the hypotheses were proved or disproved by the stochastic simulation exercise. At present, the Conclusion is too long and incudes some information that should better presented earlier on in Discussion

==============================

Please submit your revised manuscript by Jan 08 2026 11:59PM. If you will need more time than this to complete your revisions, please reply to this message or contact the journal office at plosone@plos.org. When you're ready to submit your revision, log on to https://www.editorialmanager.com/pone/ and select the 'Submissions Needing Revision' folder to locate your manuscript file.

Please include the following items when submitting your revised manuscript:

- A rebuttal letter that responds to each point raised by the academic editor and reviewer(s). You should upload this letter as a separate file labeled 'Response to Reviewers'.
- A marked-up copy of your manuscript that highlights changes made to the original version. You should upload this as a separate file labeled 'Revised Manuscript with Track Changes'.
- An unmarked version of your revised paper without tracked changes. You should upload this as a separate file labeled 'Manuscript'.

If you would like to make changes to your financial disclosure, please include your updated statement in your cover letter. Guidelines for resubmitting your figure files are available below the reviewer comments at the end of this letter.

If applicable, we recommend that you deposit your laboratory protocols in protocols.io to enhance the reproducibility of your results. Protocols.io assigns your protocol its own identifier (DOI) so that it can be cited independently in the future. For instructions see: https://journals.plos.org/plosone/s/submission-guidelines#loc-laboratory-protocols. Additionally, PLOS ONE offers an option for publishing peer-reviewed Lab Protocol articles, which describe protocols hosted on protocols.io. Read more information on sharing protocols at https://plos.org/protocols?utm_medium=editorial-email&utm_source=authorletters&utm_campaign=protocols.

We look forward to receiving your revised manuscript.

Kind regards,

Nickson Erick Otieno, PhD

Academic Editor

PLOS ONE

Journal Requirements:

When submitting your revision, we need you to address these additional requirements.

1. Please ensure that your manuscript meets PLOS ONE's style requirements, including those for file naming. The PLOS ONE style templates can be found at

https://journals.plos.org/plosone/s/file?id=wjVg/PLOSOne_formatting_sample_main_body.pdf and https://journals.plos.org/plosone/s/file?id=ba62/PLOSOne_formatting_sample_title_authors_affiliations.pdf

2. Please note that PLOS One has specific guidelines on code sharing for submissions in which author-generated code underpins the findings in the manuscript. In these cases, we expect all author-generated code to be made available without restrictions upon publication of the work. Please review our guidelines at https://journals.plos.org/plosone/s/materials-and-software-sharing#loc-sharing-code and ensure that your code is shared in a way that follows best practice and facilitates reproducibility and reuse.

3. When completing the data availability statement of the submission form, you indicated that you will make your data available on acceptance. We strongly recommend all authors decide on a data sharing plan before acceptance, as the process can be lengthy and hold up publication timelines. Please note that, though access restrictions are acceptable now, your entire data will need to be made freely accessible if your manuscript is accepted for publication. This policy applies to all data except where public deposition would breach compliance with the protocol approved by your research ethics board. If you are unable to adhere to our open data policy, please kindly revise your statement to explain your reasoning and we will seek the editor's input on an exemption. Please be assured that, once you have provided your new statement, the assessment of your exemption will not hold up the peer review process.

4. Thank you for stating the following in the Acknowledgments Section of your manuscript:

[This research was conducted as part of the USAID SERA BORA Project, contributing to the development of the Tanzania Seed Sector Development Strategy (TSSDS), which aims to enhance agricultural productivity and improve food security in Tanzania by 2030. We are grateful to ASPIRES Tanzania, supported by the USAID SERA BORA Project, for providing partial funding for this work. Thanks to Prof. David Nyange (ASPIRES) for the support that has benefited this work. Special thanks are due to the Simetar© team (www.simetar.com), particularly Dr. James W. Richardson, former Regents Professor at Texas A&M University, for his endless, invaluable guidance on the Monte Carlo Simulation Protocols.]

We note that you have provided funding information that is not currently declared in your Funding Statement. However, funding information should not appear in the Acknowledgments section or other areas of your manuscript. We will only publish funding information present in the Funding Statement section of the online submission form.

Please remove any funding-related text from the manuscript and let us know how you would like to update your Funding Statement. Currently, your Funding Statement reads as follows:

[The author(s) received no specific funding for this work.]

Please include your amended statements within your cover letter; we will change the online submission form on your behalf.

5. We note that Figure 1 in your submission contains map images which may be copyrighted. All PLOS content is published under the Creative Commons Attribution License (CC BY 4.0), which means that the manuscript, images, and Supporting Information files will be freely available online, and any third party is permitted to access, download, copy, distribute, and use these materials in any way, even commercially, with proper attribution. For these reasons, we cannot publish previously copyrighted maps or satellite images created using proprietary data, such as Google software (Google Maps, Street View, and Earth). For more information, see our copyright guidelines: http://journals.plos.org/plosone/s/licenses-and-copyright.

We require you to either (1) present written permission from the copyright holder to publish these figures specifically under the CC BY 4.0 license, or (2) remove the figures from your submission:

1. You may seek permission from the original copyright holder of Figure 1 to publish the content specifically under the CC BY 4.0 license.

We recommend that you contact the original copyright holder with the Content Permission Form (http://journals.plos.org/plosone/s/file?id=7c09/content-permission-form.pdf) and the following text:

“I request permission for the open-access journal PLOS ONE to publish XXX under the Creative Commons Attribution License (CCAL) CC BY 4.0 (http://creativecommons.org/licenses/by/4.0/). Please be aware that this license allows unrestricted use and distribution, even commercially, by third parties. Please reply and provide explicit written permission to publish XXX under a CC BY license and complete the attached form.”

Please upload the completed Content Permission Form or other proof of granted permissions as an "Other" file with your submission.

In the figure caption of the copyrighted figure, please include the following text: “Reprinted from [ref] under a CC BY license, with permission from [name of publisher], original copyright [original copyright year].”

2. If you are unable to obtain permission from the original copyright holder to publish these figures under the CC BY 4.0 license or if the copyright holder’s requirements are incompatible with the CC BY 4.0 license, please either i) remove the figure or ii) supply a replacement figure that complies with the CC BY 4.0 license. Please check copyright information on all replacement figures and update the figure caption with source information. If applicable, please specify in the figure caption text when a figure is similar but not identical to the original image and is therefore for illustrative purposes only.

The following resources for replacing copyrighted map figures may be helpful:

USGS National Map Viewer (public domain): http://viewer.nationalmap.gov/viewer/

The Gateway to Astronaut Photography of Earth (public domain): http://eol.jsc.nasa.gov/sseop/clickmap/

Maps at the CIA (public domain): https://www.cia.gov/library/publications/the-world-factbook/index.html and https://www.cia.gov/library/publications/cia-maps-publications/index.html

NASA Earth Observatory (public domain): http://earthobservatory.nasa.gov/

Landsat: http://landsat.visibleearth.nasa.gov/

USGS EROS (Earth Resources Observatory and Science (EROS) Center) (public domain): http://eros.usgs.gov/#

Natural Earth (public domain): http://www.naturalearthdata.com/

If the reviewer comments include a recommendation to cite specific previously published works, please review and evaluate these publications to determine whether they are relevant and should be cited. There is no requirement to cite these works unless the editor has indicated otherwise.

Additional Editor Comments:

For references, the guidelines for formatting were totally ignored by the authors. Several citations are missing from the list and vice versa. Richardson and kadigi are two cases in point.

Most (if not all) guidelines for manuscript formatting have been completely ignored by the authors. In assessing the revised version of the resubmitted paper, STRICT ADHERENCE TO ALL FORMATTING rules  (according to guideline documents attached here) will be the first condition for the manuscript to be considered before any further review at all, without which it will be rejected outright

Be sure to address all the above, as well as the other individual comments by reviewers, point by point in a separate document accompanying the revised paper.

[Note: HTML markup is below. Please do not edit.]

Reviewers' comments:

Reviewer's Responses to Questions

**Comments to the Author**

1. Is the manuscript technically sound, and do the data support the conclusions?

The manuscript must describe a technically sound piece of scientific research with data that supports the conclusions. Experiments must have been conducted rigorously, with appropriate controls, replication, and sample sizes. The conclusions must be drawn appropriately based on the data presented.

Reviewer #1: Partly

Reviewer #2: Yes

**********

2. Has the statistical analysis been performed appropriately and rigorously?

Reviewer #1: No

Reviewer #2: Yes

**********

3. Have the authors made all data underlying the findings in their manuscript fully available?

The PLOS Data policy requires authors to make all data underlying the findings described in their manuscript fully available without restriction, with rare exception (please refer to the Data Availability Statement in the manuscript PDF file). The data should be provided as part of the manuscript or its supporting information, or deposited to a public repository. For example, in addition to summary statistics, the data points behind means, medians and variance measures should be available. If there are restrictions on publicly sharing data—e.g. participant privacy or use of data from a third party—those must be specified.

Reviewer #1: Yes

Reviewer #2: Yes

**********

4. Is the manuscript presented in an intelligible fashion and written in standard English?

PLOS ONE does not copyedit accepted manuscripts, so the language in submitted articles must be clear, correct, and unambiguous. Any typographical or grammatical errors should be corrected at revision, so please note any specific errors here.

Reviewer #1: Yes

Reviewer #2: Yes

**********

5. Review Comments to the Author

Please use the space provided to explain your answers to the questions above. You may also include additional comments for the author, including concerns about dual publication, research ethics, or publication ethics. (Please upload your review as an attachment if it exceeds 20,000 characters)

Reviewer #1: Introduction

Why the paper deserves to publish - or contribution to the knowledge not articulated properly. "This study addresses the gap by analyzing....." not good enough without giving evidence from literature - what is the gap - then contribution to knowledge from different aspect - theory, method, practical and so on- MISSING

SDGS 2- the study deal also adaption and climate - then need to include SDG6 and 13 with the alignment of Tanzanian national policy and strategies

Methodology

Figure 1 need to daw by authors with assistance of GIS expert using ARC GIS with proper scale and others - instead of coping and paste someone work

Before Probabilistic Simulation Approach (PSA)- the statical analysis's need such as multicollinearity, VIP and other standard procedures needed

consistency between et al (italics ) or not - page 6-7

You mentioned 2 strategies only applied in this study - but you applied Multivariate Empirical (MVE) distribution???????????????????????????????????????????????????? because muti variant is Mutiple variables

Result

the socioeconomic characteristics is very important for the adoption strategy - even if not tested - need to include - in the result with the alignment of the objective

It is not clear how .. impact on food security - how measure food security - not clear from the paper

All tables and figures need sources

How measure rice productivity -not clear

Reviewer #2: Introduction - Okay

Methodology- Is it correct- The six agroecological zone

- You may exclude No fertilizer and local seed as control

Result- Okay

Discussion - Okay

Recommendation-Okay

References - Okay

**********

6. PLOS authors have the option to publish the peer review history of their article (what does this mean?). If published, this will include your full peer review and any attached files.

If you choose “no”, your identity will remain anonymous but your review may still be made public.

**Do you want your identity to be public for this peer review?**  For information about this choice, including consent withdrawal, please see our Privacy Policy.

Reviewer #1: **Yes:** Prof Yonas T. Bahta

Reviewer #2: **Yes:** Dr. Mohammad Abdullah Al Faroque

**********

[NOTE: If reviewer comments were submitted as an attachment file, they will be attached to this email and accessible via the submission site. Please log into your account, locate the manuscript record, and check for the action link "View Attachments". If this link does not appear, there are no attachment files.]

To ensure your figures meet our technical requirements, please review our figure guidelines: https://journals.plos.org/plosone/s/figures

You may also use PLOS’s free figure tool, NAAS, to help you prepare publication quality figures: https://journals.plos.org/plosone/s/figures#loc-tools-for-figure-preparation.

NAAS will assess whether your figures meet our technical requirements by comparing each figure against our figure specifications.

Attachment Submitted filename: PLOS Title paage and Affiliations Formatting Guidelines.pdf

Attachment Submitted filename: PLOSOne_formatting_sample_main_body.pdf

# Author comment: Author response to Decision Letter 1

**DOI:** 10.1371/journal.pone.0353377.r002

16 Jun 2026

The two reviewers both agree about the importance of the study, and its potential contribution to knowledge about the potential significance of improved rice production for local and national food security in a changing climate

However, they (and especially reviewer #1) feel that there are a number of technical inadequacies that undermine the quality of the paper, making it unsuitable for publication in its current state.

Comment #1

The study appears to have been pegged or relied almost entirely on analyses of rice production choice pathways (seed and fertilizer type) datasets using stochastic simulation (SS), reportedly anchored on the works of one Richardson. Although the analysis procedure is fairly well outlined, it was not very clear why the authors centered primarily on this one analytical technique and ignored all other standard strategies for assessing sociological data, which would have served to greatly complement the SS element and thus provide a more complete picture of the relative merits of fertilizer and seed choice by farmers.

Response: We thank the Reviewer #1 for this important observation. However, our primary objective in this manuscript is not to estimate the behavioral or sociological determinants of adoption (e.g., why farmers choose improved seeds or fertilizer), but rather to quantify the productivity and food-security implications (including risk and uncertainty) associated with alternative seed–fertilizer “choice pathways” observed in the National Sample Census of Agriculture (NSCA) dataset. In this context, Monte Carlo Simulation (MCS) was selected because it is particularly well-suited to (i) representing nonlinear yield risk, (ii) reproducing full yield distributions (not only mean effects), and (iii) estimating probabilities of crossing policy-relevant thresholds (e.g., 2.0 t/ha minimum and 4.5 t/ha maximum food-security yield targets).

We agree that traditional regression-type approaches (e.g., OLS/GLM) are valuable when the main aim is to estimate conditional mean effects and/or causal associations, often under parametric assumptions (normality, linearity, constant variance). However, rice yields in smallholder settings are frequently skewed, heavy-tailed, and heterogeneous across agroecological zones and management categories, and policy questions in Tanzania are commonly framed in terms of downside risk (the probability of falling below a food-security threshold) and upside opportunity (the probability of achieving high yields). MCS directly targets these probability statements by simulating from empirically grounded distributions, thereby providing an interpretable risk profile for each observed farming practice.

In addition, our dataset contains markedly unequal and sometimes small sub-samples for specific combinations of improved seed and fertilizer use (e.g., improved seed + organic fertilizer). Under such conditions, purely parametric modeling can be unstable and may require strong distributional assumptions. The probabilistic simulation approach we used (with empirical deviations/residuals and correlated sampling) is designed to preserve the observed distributional features and to generate sufficiently large simulated samples (of at least 500) for robust probability ranking, while we also validate simulated outputs against observed distributions.

That said, we fully agree with the Reviewer that complementary “traditional” analyses can enrich interpretation. To address this concern, we have strengthened the manuscript in two ways:

Clarified the study aim and method choice: We now explicitly state that the paper’s contribution is a risk-and-threshold evaluation of productivity and food security outcomes under alternative seed–fertilizer practices, rather than a behavioral adoption model [see lines 80 – 87].

Positioned complementary sociological/econometric approaches as an extension: We added a paragraph at the Conclusion section noting that adoption determinants (e.g., education, access to credit, extension services, market distance, gender, risk preferences) can be examined in a separate complementary framework (e.g., logit/probit/multinomial choice or structural models), and we identify this as an important direction for future work. This clarifies that we did not “ignore” these approaches, but rather prioritized a method aligned with our research question [see Lines 762 – 770].

What edits have we made in the manuscript?

In the Introduction: We have added a short clarification that the focus is on probabilistic productivity/food-security outcomes under observed pathways, not adoption determinants [see Lines: 80 – 87 of the revised manuscript]

In the Methodology section (particularly Section 2.3): We have added a justification paragraph explaining why MCS is appropriate versus mean-based parametric methods for threshold/risk questions and heterogeneous yield distributions [see Lines: 243 – 249].

In the Conclusion section, we have added a paragraph explaining the limitations / future research. In particular, we have stated that sociological determinants of adoption are outside the current scope and should be addressed in future complementary analysis [see Lines: 762– 770 of the revised manuscript].

***************************************************************************

***************************************************************************

Comment #2

The Introduction does not clearly articulate through literature review, what gap the research fills in knowledge on the subject, or how it advances existing knowledge based on past studies. This should entail reviewing literature up to date. The literature review should also include some introductory material on the technique adopted by the authors in their study – stochastic simulation. For instance, what are the relative merits of probabilistic/stochastic modeling for this study in comparison to other alternative analytical approaches? Furthermore, the introduction contains no literature review at all, on the relative merits of the choice or types of fertilizer and seeds, and how this relates to rural and national rice production goals/scenarios. There are tons of existing literature on that.

Response: We sincerely thank the Reviewer for this constructive and insightful comment. We agree that the original Introduction did not sufficiently (i) articulate the precise knowledge gap, (ii) position the study relative to existing empirical and modeling literature, (iii) introduce and justify the use of stochastic simulation as an analytical framework, and (iv) adequately synthesize the extensive literature on fertilizer and seed choice in relation to rice productivity and national food-security objectives.

To address this concern, we have substantially revised and restructured the Introduction to achieve four explicit objectives:

Clarify the knowledge gap

We now clearly distinguish between (a) studies that examine mean yield effects or adoption determinants of improved seeds and fertilizers, and (b) the limited number of studies that evaluate yield risk, uncertainty, and food-security threshold outcomes under alternative seed–fertilizer pathways using nationally representative data. We explicitly position our contribution as addressing this latter gap for Tanzania [see Lines 60 – 68].

Strengthen the literature review and update references

The revised Introduction now integrates additional and more relevant literature on climate change, rice productivity, seed systems, fertilizer use, and food security in sub-Saharan Africa and Tanzania, including recent contributions from Global Food Security, Nature Food, Agronomy, Ecological Economics, and FAO/World Bank policy reports. These additions complement the existing references already cited in the manuscript. These citations include, [Antle et al., 2015; Cooper et al., 2008; Godfray et al., 2010; Johnson et al., 2023; Makate et al., 2023; Mdemu et al., 2025; Nicholson, 2017; Pretty et al., 2018; Rao et al., 2015; Thornton et al., 2018; Thornton et al., 2024; Kadigi et al., 2025; Kadigi 2026].

Introduce and justify stochastic/probabilistic simulation

We added a dedicated paragraph that introduces stochastic (Monte Carlo) simulation as a well-established analytical approach for agricultural risk analysis, explicitly contrasting it with traditional mean-based econometric approaches. The revised text explains why probabilistic modeling is particularly suitable for smallholder rice systems characterized by yield variability, climatic uncertainty, and policy-relevant food-security thresholds [See Lines: 80 – 87 and Lines 69 – 81 in the introduction section, but also in the methodology section from Lines: 243 to 249].

Link seed and fertilizer choice to national and rural production goals

The revised Introduction explicitly connects fertilizer and seed choices to Tanzania’s rice development strategies, food-security objectives, and SDG 2 (Zero Hunger), showing how different input combinations relate to national productivity targets and household-level food-security outcomes [82 – 106].

These revisions substantially improve the clarity, completeness, and positioning of the manuscript and directly address the Reviewer’s concerns.

***************************************************************************

***************************************************************************

Comment #3

For such an elaborate attempt at statistical modeling, there must be at least 2 hypotheses to be tested. Not only were there no hypotheses stated or tested, there was no clear list of objectives underpinn9ng the investigation. The introduction must end with a clear list of objectives, and these should form the basis for the set of testable hypotheses for which the stochastic simulation will be run. Further down into the Discussion, include in some greater detail how you findings align with the climate adaptation concept (which you mention) within the context of United Nations sustainable development goals relevant to the subject matter

Response: We thank the Reviewer for this valuable recommendation. We agree that the manuscript will be strengthened by (i) stating clear study objectives at the end of the Introduction and (ii) formulating explicit, testable hypotheses aligned with those objectives. We also appreciate the suggestion to better situate our findings within climate adaptation framing and the UN SDGs.

At the same time, we respectfully clarify that the core contribution of our approach is probabilistic risk/threshold inference (which was the main factor in selecting this method) rather than causal hypothesis testing in the classical econometric sense. The purpose of the stochastic simulation is to reproduce yield distributions for observed farming pathways and to estimate the probability that each pathway crosses policy-relevant food-security thresholds under uncertainty. Nevertheless, we agree that the differences between simulated distributions can be framed as testable hypotheses, but since the aim of this study was to probabilistically assess the likelihood that the productivity of rice farms exceeds the required maximum thresholds or falls below the minimum values (thresholds).

Accordingly, we have revised the manuscript to:

Conclude the Introduction with explicit objectives [see Lines 97 – 108], and we have explicitly elaborated that hypothesis testing is very important, but for the nature of our study, it can be of less importance.

Expand the Discussion to explicitly interpret the results as climate adaptation evidence and link them to SDG targets [Lines: 702 – 714].

***************************************************************************

***************************************************************************

Comment #4

In methods, this study primarily involved interviews with farmers, but this element is not mentioned anywhere in the paper. Instead, all focus seems to have been given to the aspect of simulation. Stochastic simulation may have been suitable for your purpose in integrating many elements of farm management, but why were other standard procedures for assessing sociological datasets (there are numerous ones) not included to complement or supplement simulation models?

Response: We thank the Reviewer for this important clarification request and fully agree that the role of socioeconomic and sociological data should be clearly distinguished from the analytical focus of the present study.

First, we would like to clarify that this study did not involve primary interviews conducted by the authors. Instead, it relied exclusively on secondary data from the 2019/20 National Sample Census of Agriculture (NSCA), which was implemented by the Tanzania National Bureau of Statistics (NBS). The NSCA data are collected through structured household questionnaires administered by trained enumerators and include information on crop production, seed types, fertilizer use, farm size, and other household characteristics. In the revised manuscript, we have clarified this point in the Data Sources (Section 2.2) to avoid any misunderstanding that the authors conducted independent interviews [Lines: 149 – 172].

Second, regarding the absence of explicit sociological or socioeconomic modeling, we respectfully emphasize that the primary aim of this paper is not to explain farmers’ adoption behavior or socioeconomic decision-making processes, but rather to evaluate how observed seed and fertilizer practices (key policy-relevant interventions) probabilistically influence rice yield outcomes and food-security risks under uncertainty. As such, the analytical focus is deliberately placed on seed type and fertilizer application pathways as agronomic and policy levers, rather than on the socioeconomic determinants that lead farmers to choose these inputs. This scope was chosen to address a specific gap in the literature, where most studies (including Duflo et al., 2008; Kihara et al., 2026; Burke & Lobell, 2017; Stathers et al., 2020; Sheahan & Barrett, 2017; Abdoulaye et al., 2018; Mugwe et al., 2020; Khonje et al., 2015; Nin-Pratt, 2016; Vanlauwe et al., 2010), focus on adoption determinants, experimental or plot-level data, mean yield or average returns or average yield effects, while fewer quantify yield risk and food-security probabilities associated with alternative input bundles at the national scale [Lines: 60 – 67 in the Introduction section and Lines: 762 – 770 in the Conclusion section].

Third, stochastic simulation was selected because it is particularly well suited for integrating variability in farm management outcomes and for answering policy-relevant questions framed in probabilistic terms (e.g., the likelihood of exceeding or falling below food-security thresholds). While standard sociological and econometric approaches (e.g., logit/probit models, structural equation models, or mixed-methods analyses) are essential for understanding why farmers adopt certain technologies, they are less directly aligned with the study’s core research question, which concerns how different seed–fertilizer combinations translate into yield risk and food-security outcomes. Including such models in the present paper would have expanded the scope beyond its intended focus and potentially obscured the risk-based contribution of the simulation framework [Lines: 69 – 80 and Lines: 244 – 250].

That said, we fully agree with Reviewer 1’s concern that integrated studies combining socioeconomic adoption models with stochastic yield simulations are both valuable and necessary. Accordingly, we have explicitly acknowledged this in the revised manuscript by adding a paragraph in the Conclusion section as Limitations and Future Research, noting that future work could link socioeconomic determinants (e.g., education, access to credit and extension services, gender, and market access) with probabilistic simulation models to jointly analyze adoption behavior and yield-risk outcomes. This clarification underscores that socioeconomic factors are important but intentionally held outside the scope of the current analysis to maintain analytical coherence [Lines: 762 – 770].

In summary, we have made the following revisions to the manuscript:

We have clarified in Section 2.2 (Data Sources) that th

Attachment Submitted filename: Response to editors issues.docx

# Editor report: Decision Letter 1

**DOI:** 10.1371/journal.pone.0353377.r003

**Authors:** Nickson Erick Otieno

23 Jun 2026

Modeling the role of fertilizers and improved seeds on rice productivity in Tanzania using a stochastic simulation approach

PONE-D-25-39698R1

Dear Prof Kadigi,

We’re pleased to inform you that your manuscript has been judged scientifically suitable for publication and will be formally accepted for publication once it meets all outstanding technical requirements.

Within one week, you’ll receive an e-mail detailing the required amendments. When these have been addressed, you’ll receive a formal acceptance letter and your manuscript will be scheduled for publication.

An invoice will be generated when your article is formally accepted. Please note, if your institution has a publishing partnership with PLOS and your article meets the relevant criteria, all or part of your publication costs will be covered. Please make sure your user information is up-to-date by logging into Editorial Manager at Editorial Manager® and clicking the ‘Update My Information' link at the top of the page. For questions related to billing, please contact billing support.

If your institution or institutions have a press office, please notify them about your upcoming paper to help maximize its impact. If they’ll be preparing press materials, please inform our press team as soon as possible -- no later than 48 hours after receiving the formal acceptance. Your manuscript will remain under strict press embargo until 2 pm Eastern Time on the date of publication. For more information, please contact onepress@plos.org.

Kind regards,

Nickson Erick Otieno, PhD

Academic Editor

PLOS One

Additional Editor Comments (optional):

I must commend the authors most sincerely for the exceptionally excellent effort in revising the manuscript, which effort entailed meticulous point-by-point consideration and exhaustive response to all (without any exception) comments, issues and concerns raised by all the reviewers and Editor. All the responses are fully reflected in the revised and substantially improved manuscript, and all the rebuttals for points the authors felt did not warrant any changes, are very well justified and articulated.

In particular, the authors have sufficiently clarified, regarding my earlier concerns about the absence of parametrically-driven analysis and model structuring, that the study focused instead on a probabilistic approach to predicting/assessing (seed vs. fertilizer) decision pathways by farmers, and the implications of those choices for climate change and UN sustainable development goals (SDGs). They have ably and justifiably  argued that because of this analytical approach, and because, based on the nature of the datasets used in terms of skewness and variegated sample sizes and  attendant challenges in standardizing them for conventional hypothesis-driven parametric modeling, it was better to opt for stochastic simulation as a more elegant,  time-tested and defensible alternative. I am fully satisfied and convinced with this argument and its practical and technical merit.

The overall result is a highly revamped, much better structured and lucidly presented paper that should be much easier for readers to comprehend and reproduce if needed.

Based on all these facts therefore, I am satisfied that the article is acceptable for publication if the authors are willing to make the following few additional modifications

Introduction

Line 61: Please replace the word “using’ with the phrase “based on”

Line 67-68: Please consider replacing “…*avoiding crop failure or achieving yields sufficient to ensure household food security and marketable surplus* ” with “…*yield gaps or harvest shortfalls that can potentially jeopardize food security or depress projected profit margins* ”

Line 91: Your single research question should not be in italics but numbered e.g. (1). Alternatively, make it bold

Discussion

Make an even stronger highlight/emphasis in the Discussion, the implications of the findings for climate change trends in Tanzania (East Africa), and also for the UN SDGs, both of which are significant aspects of your introduction

Double-space all data in all Tables presented

Remove all figures from the manuscript body and submit them as separate TIFF, JPEG or SPS files with resolution no less than 350 dpi.  Leave only the figure captions in the relevant places in the manuscript. This should apply also to the Supporting-Information figure.

Reviewers' comments:

# Editor report: Acceptance letter

**DOI:** 10.1371/journal.pone.0353377.r004

**Authors:** Nickson Erick Otieno

PONE-D-25-39698R1

PLOS One

Dear Dr. Kadigi,

I'm pleased to inform you that your manuscript has been deemed suitable for publication in PLOS One. Congratulations! Your manuscript is now being handed over to our production team.

At this stage, our production department will prepare your paper for publication. This includes ensuring the following:

* All references, tables, and figures are properly cited

* All relevant supporting information is included in the manuscript submission,

* There are no issues that prevent the paper from being properly typeset

You will receive further instructions from the production team, including instructions on how to review your proof when it is ready. Please keep in mind that we are working through a large volume of accepted articles, so please give us a few days to review your paper and let you know the next and final steps.

Lastly, if your institution or institutions have a press office, please let them know about your upcoming paper now to help maximize its impact. If they'll be preparing press materials, please inform our press team within the next 48 hours. Your manuscript will remain under strict press embargo until 2 pm Eastern Time on the date of publication. For more information, please contact onepress@plos.org.

You will receive an invoice from PLOS for your publication fee after your manuscript has reached the completed accept phase. If you receive an email requesting payment before acceptance or for any other service, this may be a phishing scheme. Learn how to identify phishing emails and protect your accounts at https://explore.plos.org/phishing.

If we can help with anything else, please email us at customercare@plos.org.

Thank you for submitting your work to PLOS ONE and supporting open access.

Kind regards,

PLOS ONE Editorial Office Staff

on behalf of

Dr. Nickson Erick Otieno

Academic Editor

PLOS One
